# Simultaneous enhancement of stimulus-induced and stimulus-free gamma in open-eye meditators

**DOI:** 10.1162/IMAG.a.1145

**Published:** 2026-02-25

**Authors:** Ankan Biswas, Srishty Aggarwal, Kanishka Sharma, Supratim Ray

**Affiliations:** IISc Mathematics Initiative, Indian Institute of Science, Bangalore, India; Department of Physics, Indian Institute of Science, Bangalore, India; Centre for Neuroscience, Indian Institute of Science, Bangalore, India

**Keywords:** meditation, gamma oscillations, PSD slope, source localization, eLORETA, EEG

## Abstract

Visual stimuli-induced “narrowband” gamma oscillations (30–70 Hz) are generated due to excitation–inhibition interactions and are linked to attention/binding. These oscillations attenuate with aging and neurodegeneration, potentially indicating declining inhibitory function. In contrast, meditation enhances endogenous stimulus-free gamma (power in a broad frequency range above 25 Hz). However, it remains unknown how meditation affects stimulus-induced gamma or its interaction with endogenous broadband gamma, which could reveal whether meditation mitigates age-related decline in inhibitory function. We recorded electroencephalography (EEG) from long-term meditators and their healthy matched controls, performing open-eye meditation while gamma-inducing stimuli were presented before, during, and after meditation. We found that stimulus-induced gamma, like stimulus-free gamma, was stronger in meditators. Interestingly, both gamma signatures co-existed during meditation but were unrelated and prominent in occipital and fronto-temporal regions, respectively. This indicates that these two gamma signatures have different mechanisms and may be modulated differently. Reconstructed cortical sources showed meditation-induced broadband gamma localized in fronto-temporo-parietal regions, which was stronger in the parietal areas of meditators. Stimulus-induced gamma was localized to occipital areas and was stronger in meditators. Further, the power spectral density (PSD) slope, which becomes shallower with aging, was steeper for meditators. We also recorded personality traits and mystic experience using self-reported questionnaires and found that meditators were more mindful, less stressed, and had more mystical experience than the controls, but none of these scores were correlated with gamma power. Meditation could boost inhibitory mechanisms, leading to stronger gamma and steeper PSDs, potentially mitigating age-related neural changes.

## 1 Introduction

Presentation of certain visual stimuli, such as bars (Gray et al., 1989), gratings (Jia et al., 2013; Murty et al., 2018), or reddish hues (Bartoli et al., 2019; Shirhatti & Ray, 2018) induces a “narrowband” gamma rhythm in the visual cortex, which has a distinct bump in the power spectral density (PSD) of brain signals with a bandwidth of ~20 Hz and center frequency between 30 and 70 Hz. This rhythm is modulated by attention (Fries et al., 2001; Gregoriou et al., 2009) and memory (Jensen et al., 2007), linked to processes such as binding (Gray, 1999; Gray et al., 1989), gain control (Ferguson & Cardin, 2020), and normalization (Ray et al., 2013), and thought to reflect interactions between excitatory neurons and parvalbumin-positive (PV+) inhibitory interneurons (Bartos et al., 2007; Buzsáki & Wang, 2012). Recent studies have shown that the presentation of large (full-screen) stimuli induces a distinct, slower gamma between 20 and 35 Hz (termed slow gamma), which remains coherent over larger distances compared with the traditional (fast) gamma (Murty et al., 2018), and could reflect the involvement of the somatostatin inhibitory network (Chen et al., 2017; Veit et al., 2017; Wagatsuma et al., 2023). Slow gamma is in the same frequency range as beta (12–30 Hz), but it is distinct from the classical beta rhythm, which is induced in the sensory-motor areas, prominent during spontaneous periods, and suppressed by motor movements (Barone & Rossiter, 2021). Instead, slow gamma is weak or absent during spontaneous period, induced by visual stimuli, and has similar properties and spatial location as fast gamma (Murty et al., 2018). Both slow and fast gamma weaken with age (Murty et al., 2020) and are abnormal in patients suffering from brain disorders such as Alzheimer’s disease (AD) (Murty et al., 2021) and schizophrenia (Spencer et al., 2008). In addition, connectivity across brain regions reduces with the onset of AD, predominantly in slow gamma (Kumar & Ray, 2023). Together, visually evoked fast and slow gamma provide robust, well-characterized probes of cortical excitatory–inhibitory dynamics that are sensitive to healthy aging and disease.

Interestingly, gamma power is shown to be elevated in long-term meditators (Braboszcz et al., 2017; Cahn et al., 2010; Lutz et al., 2004). However, this “stimulus-free” endogenous gamma has a distinct spectral signature. It is characterized by an increase in power over a large frequency range above ~25 Hz, but there is no distinct “bump” in the PSD (Braboszcz et al., 2017). Most meditation traditions that have been studied are practiced with closed eyes or with a soft, unfocused gaze (Cahn et al., 2010; Lutz et al., 2004). Some practices incorporate visual objects as focal points for concentration, such as candles in yogic Trataka meditation (Chattha et al., 2008; Naveen et al., 1997) or complex geometric patterns in Zen practice (Kasamatsu & Hirai, 1966), but these serve primarily as aids for sustained attention and mental stabilization rather than as specific stimuli designed to systematically induce gamma oscillations. Consequently, no study to our knowledge has studied the effect of meditation on stimulus-induced gamma oscillations. Since these stimulus-induced gamma oscillations are linked to the action of different inhibitory interneuron classes (as discussed above), a comparison of stimulus-induced gamma in meditators versus age- and gender-matched controls can potentially shed light on the way meditative practices can modify the inhibitory circuitry in the brain.

A related, unresolved question is whether visually evoked narrowband gamma can co-exist with, and interact with, stimulus-free broadband gamma during meditation. Establishing their (in)dependence bears directly on mechanisms: shared variance would suggest common modulators of excitatory–inhibitory (E/I) gain (Ferguson & Cardin, 2020), whereas dissociation would support partially distinct generators and help separate trait-like broadband effects (Cahn & Polich, 2013) from state-dependent modulation of sensory gamma. However, while previous studies have characterized these gamma signatures at the sensor level ((Braboszcz et al., 2017; Lutz et al., 2004) for meditation-induced gamma; (Murty et al., 2018, 2021) for stimulus-induced gamma), scalp-level EEG alone cannot determine whether they arise from overlapping or distinct cortical generators.

To address these limitations, we recorded electroencephalography (EEG) signals from long-term (>5 years) practitioners of the Brahmakumaris (BK) Rajyoga meditation, which is uniquely performed with open eyes-enabling continuous presentation of gamma-inducing gratings during meditation—and their age- and gender-matched controls, while gamma-inducing stimuli were shown before, after, and during meditation. All meditators practiced the same standardized technique—seed-stage Rajyoga meditation—as taught by the BK organization, ensuring consistency in meditation practice across participants. Controls, who had no meditation experience, were trained in a comparable relaxation protocol involving body scanning and breath awareness (comparable with techniques used during BK meditation initiation), allowing them to engage in a focused mental practice during the experimental protocols. We also combined sensor-level analyses with cortical source reconstruction using eLORETA (Pascual-Marqui, 2007) to localize both meditation-induced broadband gamma and grating-induced narrowband gamma. This approach allowed us to test whether these gamma signatures arise from overlapping cortical networks (suggesting shared E/I modulation) or distinct generators (consistent with separable mechanisms), and whether meditators show trait-level enhancement of stimulus-induced gamma reflecting strengthened inhibitory circuits. Further, to test possible correlations between gamma power and cognitive states, we also used questionnaires to obtain their self-reported levels of mindfulness, stress, happiness, and mystic experience.

## 2 Methods

### 2.1 Participants

We recorded electroencephalogram (EEG) data from 78 participants who were either advanced meditators with at least 5 years of meditative practice (38 participants; 19 females) or control participants with no meditation experience (40 participants; 17 females), with age spanning 20–65 years, from the Indian community, predominantly from Bengaluru. To recruit healthy participants in both groups, a subjective screening was done using a health questionnaire, which included information related to any chronic or current illness, any medication, and menstrual cycle-related information for female participants at the time of experiment data collection. Also, it was ensured by self-reporting if they did not consume alcohol, tobacco, or substance of abuse in any form in the past 2 weeks.

The meditators were adept Rajyoga practitioners of the Brahmakumaris (BK) tradition who practiced meditation with open eyes. A questionnaire designed by the parent organization of the meditators was used to collect meditation practice data along with some factors to affirm the practice variables and dedication toward meditation practice and lifestyle. A top–down coordination from the headquarters of the parent organization in Mt. Abu to meditation centers in Bengaluru city was done to ensure the smooth participation of the research volunteers. Announcements regarding the study were made by the local meditation center administrators, who also verified whether the inclusion and exclusion criteria (explained below) were met. Recommended participants by local center administrators were recruited for their participation in the experiment.

Controls were recruited locally through word-of-mouth, broadcast emails, and posters after matching their age (±2 years) and gender with meditators. For female participants, we also tried to schedule the EEG recording such that the phase of the menstrual cycle was comparable with the meditator since brain oscillations in the alpha/gamma band have been shown to depend on the menstrual phase (Brötzner et al., 2014; Sumner et al., 2018). If we found a control participant first, we tried to find an appropriate meditator in a similar way. We obtained informed consent from all the participants of the study and provided monetary compensation. All procedures were approved by the institutional human ethics committee of the Indian Institute of Science, Bengaluru.

#### 2.1.1 Inclusion exclusion criteria

All participants had normal or corrected-to-normal vision and hearing, reported no history of neurological or psychiatric illness, and no current or past alcohol/substance abuse. Eligible ages were 20–70 years; individuals of either sex were included.

For the meditator group, additional criteria were verified in coordination with the Brahma Kumaris (BK) organization. Meditators were required to have ≥5 years of regular Rajyoga practice (open-eye meditation). Local BK center administrators screened prospective participants using a standardized practice-history questionnaire that recorded (i) the regularity and duration of daily sessions (morning/evening/other), (ii) lifestyle adherence commonly recommended in the BK tradition (e.g., diet, celibacy), and (iii) the approximate cumulative hours of lifetime practice. These data were used to confirm long-term, consistent engagement and to estimate total practice hours (see Section 2.5 for more details).

#### 2.1.2 Participant selection

For two meditators, there was no control participant within ±2 years, and hence, these two participants were removed. For the remaining 76 participants, we first used a fully automated pipeline (Murty & Ray, 2022) with minor modifications (described below) to find bad electrodes (Supplementary Fig. S1A) and bad stimulus repeats for each participant. We rejected 5 participants (1 meditator and 4 controls) who had more than 24 (out of 64) bad electrodes (see Section 2.6 below for more details). Therefore, the usable data consisted of 71 participants: 35 meditators (18 male and 17 female) and 36 controls (20 male and 16 female). Finally, we paired one control participant with each meditator. Pairing was done because gamma is known to be stronger in females than in males and also weakens with age in both genders (Murty et al., 2018). Pairing allows us to use paired t-tests, where the variability in gamma due to age and gender can be reduced. In the case of multiple control participants, we chose the one who had lower difference (in order of preference) in (i) age, (ii) education level, (iii) menstrual phase (for females), and (iv) EEG recording date. For two male and three female meditators, there was no control within 2 years of age difference. Therefore, out of 35 meditators, an appropriate matching control was found for 30 pairs (16 males and 14 females).

Supplementary Figure S1B shows the demographics (age, education level, days from the first day of the last menstrual cycle for non-menopausal females) and the number of bad electrodes for all 71 participants and the 30 matched pairs, along with statistical comparison using independent and paired t-tests, respectively. There was a significant difference between groups only for education level since many of the control participants were from the university campus community.

#### 2.1.3 Personality traits and measures

Self-reported data were collected using online questionnaires shared with participants before EEG recording sessions. Mindfulness score was obtained using five facet mindfulness questionnaires (FFMQ) (Baer et al., 2006), stress score using the perceived stress scale (Cohen et al., 1983), happiness score using the Oxford happiness questionnaire (Hills & Argyle, 2002), while mystic experience score using mystic experiences questionnaire (Barrett et al., 2015).

### 2.2 EEG recordings

EEG signals were recorded from 64 active electrodes (actiCAP) using the BrainAmp DC EEG acquisition system (Brain Products GmbH). The electrodes were placed according to the international 10–10 system, and the signals were referenced to FCz during acquisition. Raw signals were filtered online between 0.016 Hz (first-order filter) and 250 Hz (fifth-order Butterworth filter), sampled at 1000 Hz, and digitized at 16-bit resolution (0.1 μV/bit). The participants were asked to sit in a dark room in front of a gamma-corrected LCD monitor (BenQ XL2411; dimensions: 20.92 × 11.77 inches; resolution: 1280 × 720 pixels; refresh rate: 100 Hz), placed at ~58 cm from the participants and subtended 52° × 30° of visual field for full-screen gratings. Eye position was monitored using EyeLink Portable Duo head-free eye tracker (SR Research Ltd, sampled at 1000 Hz).

### 2.3 Experimental procedures

**Fig. 1.**
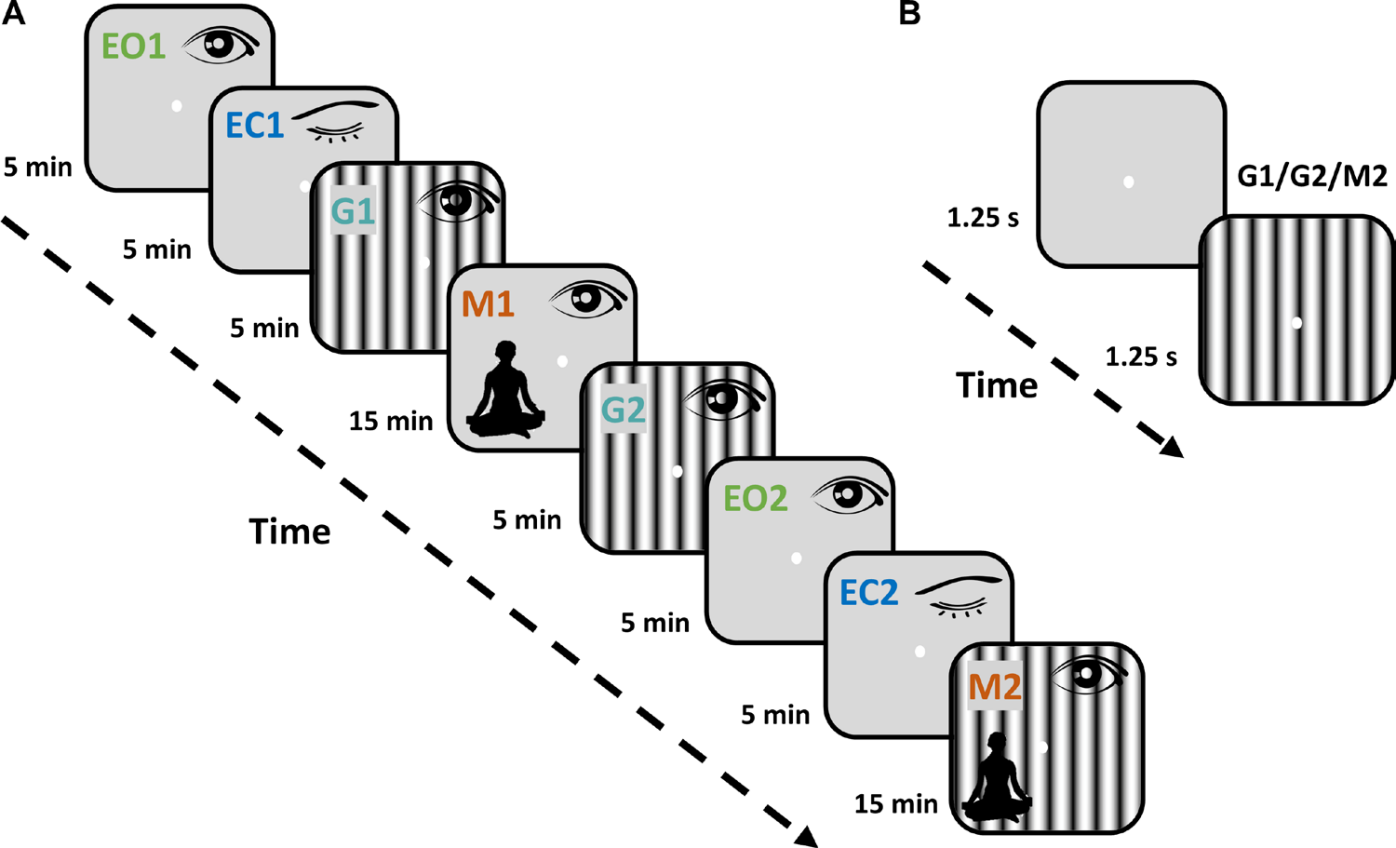
Experimental Design and the Task Paradigm (A) The experiment consisted of eight protocols, starting with eyes-open (EO1) condition where participants were required to maintain fixation on a dot on the screen. The eye position of the participants was monitored using a restraint-free eye tracker for all protocols where eyes were open, and epochs where the eye position deviated beyond 2.5 degrees from fixation were discarded offline later. EO1 was followed by the eyes-closed (EC1) condition. Gamma protocols (G1 and G2) were conducted before and after the stimulus-free meditation protocol (M1) where participants were asked to meditate while keeping their eyes open. The experiment was concluded with another set of eyes-open (EO2) and eyes-closed (EC2) protocols, followed by a second meditation protocol where visual stimuli were shown while participants meditated keeping their eyes open (M2). Respective session duration in minutes is indicated beside the corresponding rectangular box for every protocol. (B) Participants were asked to look at a dot at the center of the screen in all protocols except EC1 and EC2. For G1, G2, and M2 protocols, gamma-inducing achromatic gratings (spatial frequency of 2 and 4; orientation of 0, 45, 90, 135) were presented for 1.25 seconds with an inter-stimulus interval of 1.25 seconds in a continuous sequence. The participants were asked to blink or break fixation during the inter-stimulus interval if needed.

The experiment consisted of eight protocols, as shown in Figure 1. It started with a passive fixation on a center dot on the screen (EO1), followed by the eyes closed condition (EC1), further followed by the passive visual fixation task (G1) where the full-screen grating stimulus was presented for 1.25 seconds with an interstimulus interval of 1.25 seconds, with each protocol lasting ~5 minutes. Stimuli were presented in an uninterrupted sequence, and participants were asked to blink (if needed) during the inter-stimulus period. Next, participants were asked to meditate with open eyes for ~15 minutes while keeping their gaze on the screen (M1). The next three protocols were repeats of the first three protocols (G2, EO2, and EC2). In the last protocol (M2), participants were asked to meditate with open eyes while full-screen grating stimuli were presented (like G1 and G2 protocols) for ~15 minutes. The gamma-inducing achromatic grating stimuli had a spatial frequency of 2 or 4 cycles per degree and orientation of 0°, 45°, 90°, 135° (total 8 combinations). Even for eyes open (EO1 and EO2), eyes closed (EC1 and EC2), and stimulus-free meditation (M1) conditions, where the fixation spot remained on throughout the protocol, we segmented the data into 2.5 second “trials” involving 1.25 seconds of “spontaneous” and 1.25 seconds of “stimulus” periods to have a uniform trial structure across protocols (for protocols where no stimulus was presented, we averaged the PSDs of the two epochs). All protocols had 120 trials except for M1 and M2, which had 360 trials.

### 2.4 Meditation technique

#### 2.4.1 Meditators

The meditators practiced Rajyoga meditation (seed-stage meditation) of the BK tradition, which is practiced with open eyes. The practice employs contemplation and directed thinking in order to reach experiential states for self-development (Nair et al., 2017). The practice utilized in the current study was focused on shifting the awareness from the visible world to “the soul and its peaceful nature” and then connecting to the “Supreme Soul”. While practicing the seed stage, meditators started with the awareness of their body and slowly shifted their focus to the middle or behind the forehead and imagined themselves as a point of light situated in the body. This step is known as “soul consciousness” and is common to all practices in the BK Rajyoga tradition (Hassija, 1978; Nair et al., 2017; Sharma et al., 2023). In the final stage, meditators connect with the Supreme Soul, imagine a conversation with the Supreme Soul, and establish a connection. Meditators affirm and reflect on seed-like qualities of the soul (peace, power, bliss, love, purity, and wisdom) and imagine receiving these qualities from the Supreme Soul, which permeates the meditator’s soul. On a successful connection, a feeling of completeness emerges. A feeling of detachment from the physical world emerges, and meditators experience themselves in complete silence and subtleness. Seed stage symbolizes attaining original seed-like qualities seen as spiritual treasures that need to be awakened and nurtured. Meditators mentally repeat positive statements in affirmation to internalize and imbibe these qualities, such as “I am a peaceful soul,” “I am a pure soul,” and so on. Meditators with repeated practice can switch to the seed stage in minimum time and remain in soul consciousness as a precursor.

#### 2.4.2 Controls

To practice relaxation beforehand, control participants were asked to follow audio instructions. The instructions for relaxation intervention were recorded in a male voice in both English and Hindi languages, given to the recruited participant in the form of audio files and asked to practice at least once before the experimental EEG recording. Starting with body scanning, participants were asked to focus on different body areas, starting from the toe of both feet and gradually moving to other parts of the body and ending at the tip of the head. Apart from body scanning, they were asked to focus on the breath and sync it with the body scanning with the instruction that “every incoming breath is energizing your body, and every outgoing breath is relaxing it”. After this, they were asked to focus on the middle of the forehead and imagine a point of light. This procedure is similar to the technique used by BK meditators during initiation of their meditation practice.

We note that the pre-meditation instructions for meditators and controls differ slightly, which may limit comparability. For example, unlike the control group, we chose not to provide any explicit instructions to the meditators because we did not want such instructions to interfere with their meditation experience (they did receive comparable instructions during their initial training in the BK Rajyoga tradition). This could lead to some physiological differences or additional memory load when recalling the instructions. Further, it cannot be ascertained that controls could reproduce the meditation without the audio recording.

To ensure that the meditation technique was as comparable between the groups as possible, we followed up with controls before they came for the EEG recording via phone calls and messages to verify that they understood the relaxation technique and could successfully follow the protocol. This follow-up ensured that all control participants were adequately prepared and comfortable with the relaxation procedure before participating in the study. During the EEG recording, both groups practiced meditation, and we did not provide any audio instructions to either group. This ensured comparable sensory conditions while allowing participants to engage meaningfully in their respective practices.

### 2.5 Meditation experience

The meditation practice data were also collected in terms of daily meditation practice minutes in the morning, evening, and any other time of the day, weekly practice, and years since they started practicing it. Based on these, the total number of hours of meditative practice was calculated for all meditators except two for whom this information was incomplete. Overall, the meditators had (mean ± standard deviation (SD)) 10651 ± 7717 (n = 33; min: 2097, max: 30576) hours of practice.

### 2.6 Artifact rejection

We removed artifacts in the data using the following criteria (adapted from Murty & Ray, 2022). We rejected electrodes with an impedance greater than 25 kΩ. The impedance of the final set of electrodes was (mean ± SD) 6.19 ± 4.80 kΩ. Further, for each electrode, we detected outliers as trials with root mean square (RMS) outside 1.5–35 μV or with deviation from the mean signal in the frequency domain by more than 6 SD, and subsequently, we discarded the electrodes with more than 30% outliers. Further, we considered trials that were deemed bad in the occipital electrode groups or in more than 10% of the other electrodes as bad, eventually obtaining a set of common bad trials for each protocol for each participant. Overall, this led us to reject (mean ± SD) 17.2±6.9%, 19.2±7.0%,  16.8±5.5%,  23.8±7.4%,  17.7±6.5%,  18.5±  7.9%, 19.8 ± 8.2%, and 23.1 ±  8.0% trials in EO1, EC1, G1, M1, EO2, EC2, G2, and M2, respectively. Next, we computed slopes for the power spectrum between 56 and 84 Hz range for each unipolar electrode and rejected electrodes whose slopes were less than 0. We found that electrodes Fp1, FC2, FC3, FC4, FC5, FC6, FT7, TP8, and C2 (indicated in Supplementary Fig. S1A) were bad in more than 35% of the participants either in the meditator or control group and hence, we excluded them for analysis in all participants. Finally, we rejected participants who had more than 35% bad electrodes. This led to a rejection of five participants (one meditator and four controls; Supplementary Fig. S1A).

#### 2.6.1 Eye artifacts

We declared eye blinks or changes in eye position outside a 5° fixation window (i.e., ± 2.5° from the fixation spot after correcting for offset from the center fixation spot) during −1 to 1.25 seconds from stimulus/marker onset as fixation breaks and removed them offline. This led to a rejection of a total of (mean ± SD) 31.9±21.1%, 22.2±19.9%, 39.0± 27.3%, 25.7±21.1%, 33.0±23.1%, and 26.2±20.0% trials in EO1, G1, M1, EO2, G2, and M2, respectively. This relatively higher proportion of fixation breaks compared with our previous studies (Murty et al., 2020) is because of the longer analysis duration (-1 to 1.25 seconds compared with -0.5 to 0.75 seconds in previous studies) and also because the stimuli were presented in a continuous manner here as opposed to a trial-wise manner with longer inter-trial intervals in previous studies. We also repeated all analyses without removing bad eye trials and found that results were similar with or without the removal of bad eye trials.

For each protocol, we rejected participants with less than 30 good trials and 3 good electrodes in each electrode group and for each epoch (stimulus or baseline). This led to a rejection of one participant in EO1, EC1, G1, M1, M2 and two participants in G2 for the occipital electrode group, and one participant in EO1, G1, M1 and two participants in G2 for the fronto-temporal electrode groups. Therefore, we had 30 pairs for EC1 fronto-temporal group and 29 paired participants for all remaining comparisons except G2, for which we had 28 pairs for the analysis using both the electrode groups. For comparison across protocols (say M1 vs. EO1), we selected participant pairs that were good for both protocols, leading to a further reduction in the number of pairs for some comparisons (the number of matched pairs varied between 27 and 30 for all comparisons).

### 2.7 EEG data analysis

All the data analyses were done using custom codes written in MATLAB (MathWorks. Inc; RRID: SCR_001622). The analyses were performed using unipolar reference scheme. We chose two groups of electrodes, (i) fronto-temporal: a combination of frontal and temporal electrodes (Fp1, Fp2, AF3, AF4, AF7, AF8, F5, F6, F7, F8, FC5, FC6, FT7, FT8, T7, T8, C3, C4, C5, C6, TP7, TP8) to depict the effect of meditation and (ii) occipital: a combination of parieto-occipital and occipital electrodes (P3, P1, P2, PO3, POz, PO4, O1, Oz and O2) for which strong stimulus-induced gamma was observed. Scalp maps were generated using the topoplot function of EEGLAB toolbox (Delorme & Makeig, 2004) (RRID:SCR_007292) with standard Acticap 64 unipolar montage of the channels.

#### 2.7.1 Power analysis

Power spectral density (PSD) and the time–frequency power spectrogram were obtained using multi-taper method with a single taper using the Chronux Toolbox (Bokil et al., 2010) (RRID: SCR_005547) for individual trials and then averaged across the trials for each electrode. For G1, G2, and M2, we chose “baseline” or “spontaneous” period between -1 and 0 seconds of stimulus onset, while stimulus period between 0.25 and 1.25 seconds to avoid stimulus onset-related transients, yielding a frequency resolution of 1 Hz for the PSDs. For uniformity, for other protocols where there was no stimulus (EO1, EC1, M1, EO2, and EC2), we put imaginary stimulus markers at 2.5-second intervals, chose baseline and (imaginary) stimulus periods as before, and then averaged the PSDs across baseline and stimulus. The spectrograms were obtained using a moving window of size 0.25 seconds and a step size of 0.025 seconds, thus yielding a frequency resolution of 4 Hz. Note that spectrograms were only shown for completeness—all power calculations and subsequent statistical tests were done based on the PSDs, which had a frequency resolution of 1 Hz.

We have previously shown that full-screen gratings induce two distinct gamma oscillations, which we termed slow and fast gamma (Murty et al., 2018). Although the peak gamma frequencies vary across individuals and also slow down with age for both gamma bands (Murty et al., 2020), we chose fixed frequency bands for analysis to minimize free parameters in the power calculation. Since this dataset consists of younger participants compared with our previous dataset, we chose a slightly higher frequency of 24–34 Hz for stimulus-induced slow gamma (in previous studies, we had participants between 50 and 88 years and used 20–34 Hz; Murty et al., 2020, 2021). The stimulus-free gamma range was chosen between 30 and 80 Hz. We calculated the change in power in the chosen frequency ranges as

$$\Delta Power=10\left(\log_{10}\frac{\sum PSD_{P2}\left(f\right)}{\sum PSD_{P1}\left(f\right)}\right),$$

where P2 and P1 are the epochs or protocols.

Stimulus-induced slow gamma was defined as 24–34 Hz based on the power spectral density (PSD) across all analyses. In the M2 (meditation-with-stimulus) protocol, where meditation elevated the baseline PSD, we also tested 20–30 Hz range.

#### 2.7.2 Slope analysis

We computed the slope of the 1/f aperiodic component of the spectral distribution for frequency range of 104–190 Hz using the MATLAB wrapper for Fitting Oscillations and One Over f (FOOOF) toolbox (Donoghue et al., 2020). The settings chosen for FOOOF model parameters were peak width limits: [4 8]; the maximum number of peaks: 5; minimum peak height: 0.2; peak threshold: 2.0; and aperiodic mode: “fixed”, as used previously (Aggarwal & Ray, 2023). We discarded slopes less than 0.1, which occurred due to poor fitting.

#### 2.7.3 Source localization

For source localization, we followed the same source localization methodology using eLORETA that we used in our previous study (Biswas et al., 2025). Briefly, we estimated sources using the eLORETA technique using fieldtrip toolbox (Oostenveld et al., 2011) in MATLAB (MathWorks Inc., RRID: SCR_001622). We started with pre-processing the EEG time series data where bad electrodes were interpolated, data were average referenced, and trials were segmented into baseline and stimulus epochs. The cleaned data were transformed into the frequency domain using multitaper spectral analysis, which was used to compute the cross-spectral density matrix that is required to perform source localization directly in the frequency domain (see Biswas et al., 2025 for more details). We performed source localization for two distinct gamma frequency ranges: broadband endogenous gamma (55–80 Hz) and stimulus-induced gamma (24–34 Hz). This broadband range was used to avoid the line noise at 50 Hz; similar results were obtained when we used 30–45 Hz for analysis. A standard MRI-based head model was created using a boundary element method (BEM), and electrodes were co-registered for accurate alignment. A lead field matrix was computed for a 5-mm grid source model. Source activity was estimated using eLORETA, a weighted minimum norm approach with depth regularization, and visualized to map brain oscillatory activity.

### 2.8 Statistical data analysis

Since our data were matched and paired across participants in the two groups, we compared means of the PSDs, change in power, and slopes between the groups using paired t-test. Also, we verified the results by comparing the full dataset of 35 meditators and 36 controls using independent-samples t-test. By pairing age- and gender-matched participants, variability in gamma power solely due to age- and gender-related differences was reduced, because in paired t-test, we compute the difference in power between the pairs and test whether this difference is significantly different from zero. However, we found that independent-samples t-tests yielded similar results, potentially due to slightly larger sample sizes (see Supplementary Table S1). We evaluated correlations between variables using both Pearson’s correlation (*r*) and Spearman’s rank correlation (*ρ*). Pearson’s *r* was computed using MATLAB’s “corrcoef” function, while Spearman’s *ρ* was computed using the “corr” function. Spearman’s *ρ* was included to capture potential monotonic but non-linear dependencies. All correlations were computed separately for meditators and controls. We performed voxel-wise paired two tailed t-tests to compare source power between groups (meditators vs. controls), protocols (e.g., M1 vs. EO1), and time intervals (stimulus vs. baseline), with resulting t-values generating statistical parametric maps (t-maps) that visualize spatial distributions of significant differences. We set the significance threshold at p < 0.05 (uncorrected) for display purposes. We did not apply any correction for the display to better show the extent of power changes across the cortex. The uncorrected t-statistic maps provide a continuous representation of effect strength, from which p-values are directly derived.

#### 2.8.1 False discovery rate correction

The primary statistical analyses in this study were hypothesis driven and restricted to predefined frequency bands and anatomically defined electrode clusters (fronto-temporal and occipital), based on prior evidence that long-term meditation enhances gamma activity in specific cortical regions (Braboszcz et al., 2017; Cahn et al., 2010; Lutz et al., 2004). Because these comparisons were limited in number and grounded in a priori hypotheses, broad multiple-comparison correction was not required. This rationale follows established methodological guidelines distinguishing confirmatory (hypothesis-driven) from exploratory analyses, where correction is warranted only when many independent tests are conducted (Nichols & Hayasaka, 2003; Poldrack et al., 2017).

To control for multiple comparisons in the broader descriptive analyses—particularly when reporting the statistical summary tables—we applied the Benjamini–Hochberg procedure (Benjamini & Hochberg, 1995) for False Discovery Rate (FDR) correction. FDR correction was implemented at two distinct levels: (1) table-wide, considering all p-values across all frequency bands simultaneously, and (2) row-wise, applying FDR correction independently within each frequency band. The table-wide approach provides protection against false positives across the entire set of comparisons, while the row-wise approach offers increased sensitivity for detecting significant effects within specific frequency bands. In these tables, significant p-values (p < 0.05) without any correction are marked with an asterisk (*), while those after row-wise FDR correction are marked with “†”. Finally, those significant after table-wide correction are shown in bold. Row-wise FDR correction was only applied when a frequency band contained at least two p-values, as FDR correction is not meaningful for a single test.

## 3 Results

We analyzed EEG data from 71 participants (35 long-term meditators and 36 matched controls) who successfully completed all experimental protocols (Fig. 1). Each session consisted of eight sequential conditions alternating between eyes-open and eyes-closed rest, visual stimulation, and open-eye meditation. Participants first performed eyes-open fixation (EO1) and eyes-closed rest (EC1), followed by a gamma-inducing visual task (G1). They then engaged in stimulus-free open-eye meditation (M1), after which the sequence was repeated (G2, EO2, EC2). Finally, participants performed a second meditation block (M2) during which the same visual stimuli were presented. This design allowed us to directly compare brain activity across rest, meditation, and visually driven conditions, both before and after meditation.

EEG data were recorded from 64-channel active electrode caps; only participants with at least 40 good electrodes were retained for analysis (see Supplementary Fig. S1 for demographic and quality metrics). To minimize variance due to known effects of age and gender on gamma power, each meditator was paired with a control participant within 2 years of age and of the same gender. Statistical analyses were, therefore, conducted using paired t-tests, with independent-samples t-tests yielding similar outcomes (see Supplementary Table S1). For clarity and physiological interpretability, we focused on two anatomically defined electrode clusters: occipital sites, which show strong stimulus-induced gamma during visual stimulation, and fronto-temporal sites, where meditation-related broadband gamma and slope effects were most prominent.

### 3.1 Meditators have more stimulus-free gamma during eyes-open and eyes-closed conditions

**Fig. 2.**
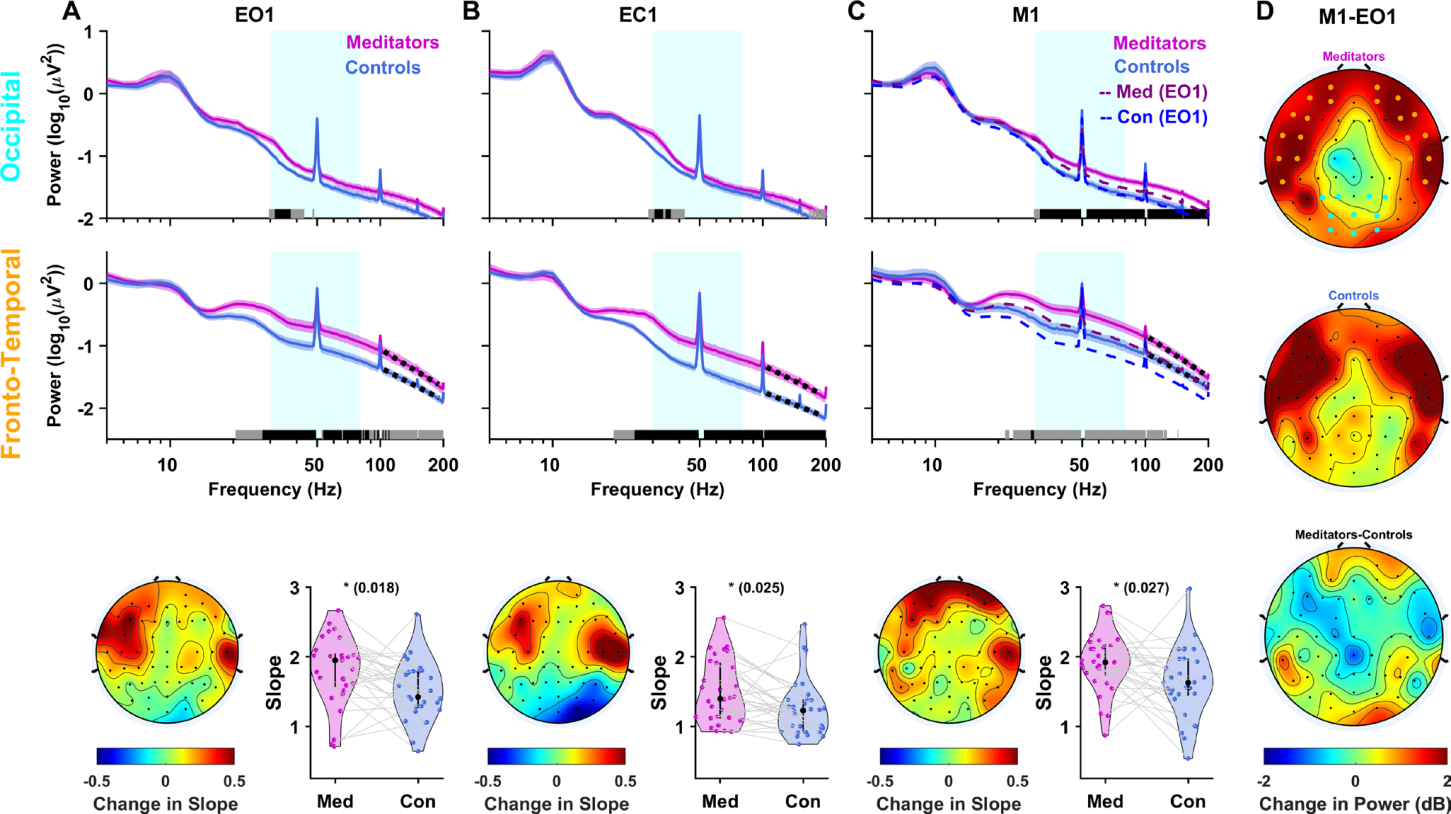
Meditators have more stimulus-free gamma during eyes-open, eyes-closed, and meditation conditions. (A) The top two rows show the power spectral density (PSD) averaged across 29 meditators (magenta) and their matched controls (blue) for the EO1 protocol for the occipital and fronto-temporal electrode groups, respectively. Solid traces represent the mean and the shaded region around them indicates standard error of the mean (SEM) across participants. Horizontal bars at the abscissa represent the significance of differences in mean (gray: p < 0.05 and black: p < 0.01, paired t-test, not corrected for multiple comparisons). In the bottom panel, the average scalp maps (topoplots) of the 64 electrodes on the left show the change in slope between meditators and controls (meditators–controls), computed between 104 and 190 Hz (highlighted as black dotted lines over PSDs in the middle panel). The violin plot on the right shows the mean slope for meditators and controls in the fronto-temporal electrode group. Means are compared using paired t-test, and the p-values are indicated at the top, along with their significance level (*: p < 0.05, **: p < 0.01, ***: p < 0.005). (B) PSD and slope plots for the EC1 protocol when the eyes were closed. n = 29 for top row and n = 30 for middle and bottom rows. (C) PSD and slope plots when participants meditated keeping their eyes open in the M1 protocol (n = 29). Dashed lines show PSDs for the EO1 protocol for the meditators and controls (same plots as shown in A) for comparison. (D) Topoplots show change in gamma power (in decibels (dB)) during M1 compared with EO1 for meditators (top) and controls (middle) (n = 28). The bottom plot shows the change in power in M1 relative to EO1 between meditators versus controls (difference between the top and the middle row).

Figure 2A shows the mean PSDs for meditators and their age- and gender-matched controls (n = 29) for the EO1 segment for the occipital (top row) and fronto-temporal electrode groups (middle row). The sharp peaks at 50 Hz and 100 Hz are artifacts due to line noise and harmonics. Even when not meditating, meditators had more power than controls over a broad frequency range above ~20 Hz, with a shallow “bump” in the PSD around ~30 Hz that was more prominent for meditators. This stimulus-free “broadband” gamma power (calculated between 30 and 80 Hz) was significantly higher in meditators for all electrodes (Supplementary Fig. S2A), but more prominent in fronto-temporal regions (t_(28)_ = 3.18, p = 0.004; Supplementary Fig. S3A). Table 1 gives the same analysis done in traditional bands across all protocols, which shows that the difference in power between meditators and controls was more prominent in the gamma range. Supplementary Table S1 gives the same results using independent t-tests on the full (unpaired) dataset. These tables also indicate results after doing correction for multiple comparisons, as discussed in Section 2.

**Table 1.** P-values and statistics of the t-tests performed between paired meditators and controls for different frequency bands for all the protocols for occipital and fronto-temporal electrodes.

	**Band**		**Protocols**								
**Occipital group**			**EO1 (29)**	**EC1 (29)**	**G1 (29)**	**M1 (29)**	**G2 (28)**	**EO2 (25)**	**EC2 (29)**	**M2_bl (29)**	**M2_st (30)**
	Delta (1–3 Hz)	t =	1.83	1.38	0.01	0.75	0.76	-0.13	0.09	-1.53	-1.48
		p =	0.077	0.178	0.996	0.462	0.452	0.899	0.93	0.138	0.15
	Theta (4–7 Hz)	t =	1.16	0.66	0.76	0.23	-0.31	-0.33	0.37	-0.55	-0.09
		p =	0.255	0.514	0.451	0.816	0.762	0.747	0.714	0.585	0.933
	Alpha (8–12 Hz)	t =	0.1	0.28	0.79	0.63	0.61	-0.48	-0.23	-0.19	-0.25
		p =	0.917	0.781	0.437	0.531	0.548	0.633	0.822	0.847	0.805
	Beta (13–23 Hz)	t =	0.92	0.3	1.75	0.21	0.81	0.82	0.59	0.45	0.9
		p =	0.366	0.763	0.09	0.833	0.424	0.42	0.563	0.656	0.375
	Slow gamma (24–34 Hz)	t =	1.92	2.13	2.26	1.87	2.98	1.22	2.76	2.47	3.03
		p =	0.066	*0.042	*0.032	0.071	†0.006	0.234	†0.01	†0.0199	†0.005
	Broadband gamma (30–80 Hz)	t =	2.33	2.38	0.49	3.32	1.87	0.86	3.47	4.26	4.57
		p =	†0.027	†0.024	0.627	**†0.003**	0.073	0.398	**†0.001**	**†2** **×** **10^-4^**	**†1** **x** **10^-4^**
**Fronto-temporal electrode group**			**EO1 (29)**	**EC1 (30)**	**G1 (29)**	**M1 (29)**	**G2 (29)**	**EO2 (26)**	**EC2 (30)**	**M2_bl (30)**	**M2_st (30)**
	Delta (1–3 Hz)	t =	1.76	0.64	1.3	0.01	-0.08	-0.94	-0.2	0.86	0.96
		p =	0.089	0.529	0.204	0.992	0.938	0.354	0.843	0.395	0.345
	Theta (4–7 Hz)	t =	1.06	0.55	0.9	-0.51	0.64	-2.15	-0.37	0.31	0.63
		p =	0.298	0.588	0.376	0.617	0.529	*0.041	0.717	0.757	0.534
	Alpha (8–12 Hz)	t =	0.44	0.6	1.72	-0.92	0.02	-1.32	-0.36	-0.71	-0.84
		p =	0.666	0.555	0.096	0.366	0.985	0.199	0.719	0.481	0.405
	Beta (13–23 Hz)	t =	1.72	1.57	0.54	1.27	0.33	-0.59	1.31	2.55	2.79
		p =	0.097	0.127	0.593	0.216	0.748	0.558	0.2	†0.016	**†0.009**
	Slow gamma (24–34 Hz)	t =	3.32	4.42	-0.73	2.5	0.15	0.86	3.28	4.11	4.27
		p =	**†0.002**	**†1** **×** **10^-4^**	0.47	†0.019	0.879	0.396	**†0.003**	**†3** **×** **10^-4^**	**†2** **×** **10^-4^**
	Broadband gamma (30–80 Hz)	t =	3.18	4.9	0.01	2.48	-0.56	0.9	3.08	4.35	4.4
		p =	**†0.004**	**†1** **×** **10^-5^**	0.996	†0.019	0.58	0.377	**†0.005**	**†2** **×** **10^-4^**	**†1** **×** **10^-4^**

For G1 and G2 protocols, we have used change in power during the stimulus period from the baseline. For other protocols, we have used raw power for the comparison between the two groups. For M2 protocol, we have separately done the comparison for the baseline (bl) and stimulus period (st) using raw power. P-values with * in front of them highlight the p-values for which result was significant (p < 0.05) without any correction between the two groups. ^†^Symbol indicates values that remained significant after row-wise FDR correction (i.e., nine entries). Bold p-values remained significant after FDR correction for the individual table for each electrode group (54 entries). For each protocol, the total number of participants (n) used in the t-tests is shown in parentheses after protocol names, with degrees of freedom (df) calculated as n − 1 for each respective protocol.

### 3.2 The slope of the power spectral density is steeper for meditators than controls

We also compared the slope of the PSD between meditators and controls since PSD slopes become shallower with age (Voytek et al., 2015) and steeper slopes have been associated with higher inhibition (Gao et al., 2017). Slopes for meditators were indeed steeper than controls over the fronto-temporal electrodes, albeit over a higher frequency range (104–190 Hz; dotted black lines in the middle row) than used previously (Voytek et al., 2015). As shown in the scalp maps of the difference in slope between meditators and controls (Fig. 2A, bottom row; t_(28)_ = 2.518, p = 0.018), this steeping of the slope was mainly observed in the fronto-temporal regions.

Both the broadband change in power and the steepening of slope in fronto-temporal regions were observed when eyes were closed (EC1; Fig. 2B and Supplementary Fig. S3B; power: t_(29)_ = 4.9, p = 1 x 10^-5^; slope: t_(29)_ = 2.361, p = 0.025; also see Supplementary Fig. S2B), ruling out potential confounds related to myogenic activity related to open eyes.

### 3.3 Meditation increases broadband gamma over fronto-temporal areas for controls, and across all areas in meditators

Stimulus-free meditation increased broadband gamma power (Fig. 2C; compare the PSDs for M1 with EO1 shown in dashed lines) in both meditators and controls (n = 29). However, in occipital areas, this increase was mainly observed for meditators (Fig. 2C, top row; the separation between solid and dashed lines is only observed for meditators), while both meditators and controls showed an increase in fronto-temporal areas (Fig. 2C, middle row). To better visualize the effect of meditation across brain regions, we plotted scalp maps for the broadband gamma power (30–80 Hz) during M1 relative to the power in the EO1 segment (Fig. 2D; n = 28; this is obtained by subtracting the power for the two conditions on a log scale and multiplying by 10 to have units of decibels (dB)). Meditators showed an increase in both occipital and fronto-temporal areas (Fig. 2D, top row). In contrast, for controls, the increase was mainly in the fronto-temporal electrodes (Fig. 2D, middle row). This meditation-induced increase in power during M1 compared with EO1 in meditators versus controls was observed mainly in occipital and frontal regions (Fig. 2D, bottom row).

The slopes for meditators were significantly steeper than controls even during M1 (bottom row of Fig. 2C, t_(28)_ = 2.336, p = 0.027). While the slopes during M1 were also slightly higher than EO1 for both groups, the difference did not reach significance (meditators: n = 28 (mean ± SEM) EO1: 1.80 ± 0.09, M1: 1.89 ± 0.08, p = 0.12, t-test; controls: n = 28, EO1: 1.50 ± 0.08, M1: 1.63 ± 0.1, p = 0.07, t-test).

The power and slope distributions sometimes had a non-Gaussian appearance (e.g., EC1 slopes for meditators appear to be double Gaussian), which could be due to heterogenous response within the meditators group. Therefore, we also used non-parametric tests to compare power and slopes between meditators and controls. For power, the comparison using Wilcoxon sign-rank test for paired comparison is summarized in Supplementary Table S2, while comparison using Mann–Whitney U test for unpaired samples is summarized in Supplementary Table S3. For the comparison of slopes shown in Figure 2 (bottom plots), the corresponding p-values for EO1, EC1, and M1 using Wilcoxon sign-rank test are 0.027, 0.016, and 0.021. Therefore, all key results remained significant using both parametric and non-parametric tests.

### 3.4 Stimulus-induced gamma is stronger for meditators even in non-meditative condition

**Fig. 3.**
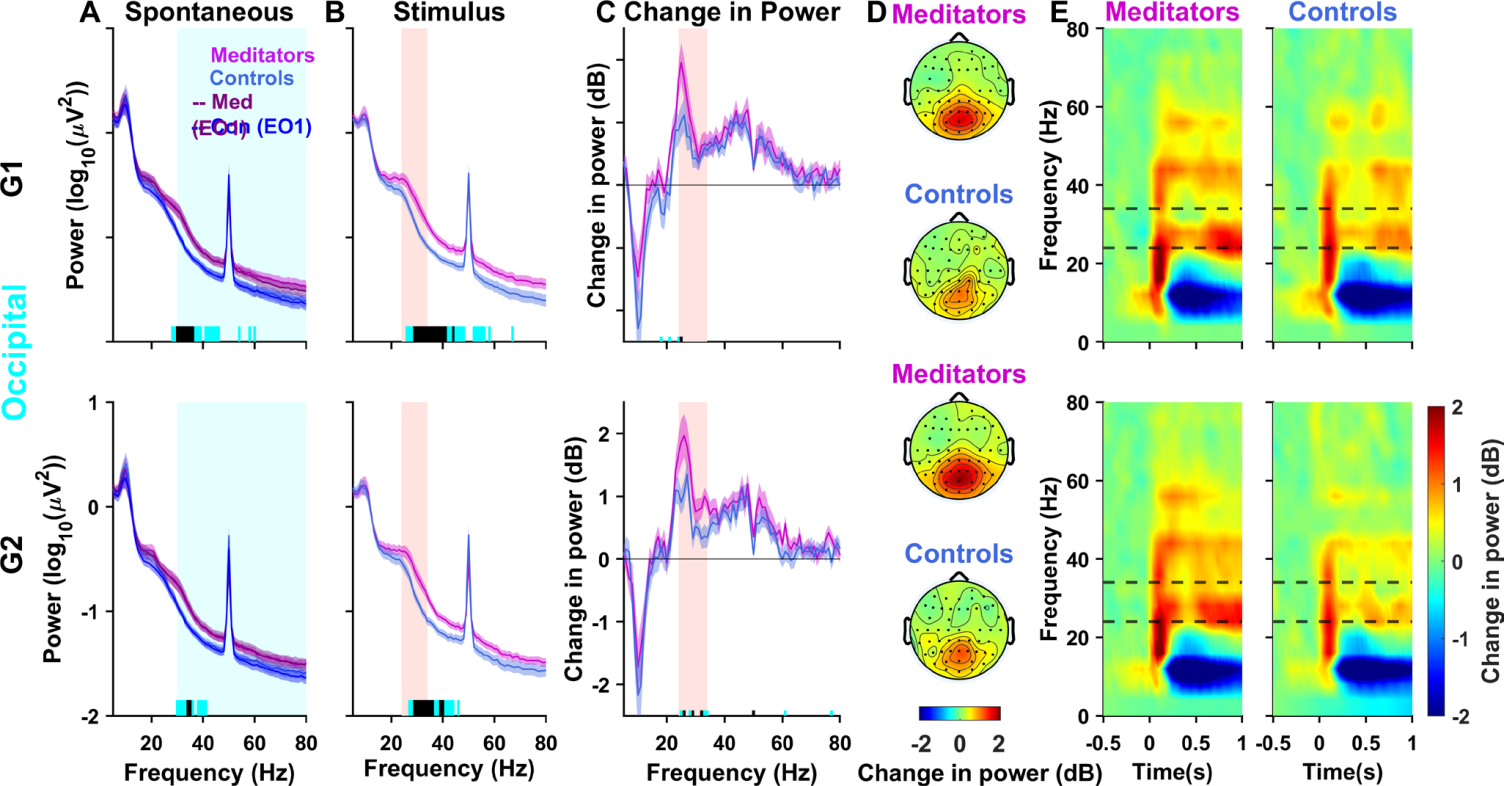
Stimulus-induced “slow” gamma is stronger for meditators. (A) Average PSDs across paired participants for the occipital electrode group for the spontaneous (baseline) period (-1 to 0 seconds) before the stimulus onset for the meditators (magenta) and controls (blue) for G1 (top panel; n = 29) and G2 (bottom panel; n = 28) protocols. Solid traces represent the mean, and shaded regions around them indicate SEM across participants. Dashed lines show corresponding PSDs for the EO1 protocol. Horizontal bars at the abscissa represent the significance of differences in mean (gray: p < 0.05 and black: p < 0.01, paired t-test). (B) Average PSDs calculated for the “stimulus” period (0.25 to 1.25 seconds from the stimulus onset) for the respective protocols. (C) Change in power in dB during the stimulus with respect to the baseline for the G1 (n = 29) and G2 (n = 27) protocols. (D) Topoplots for meditators (top) and controls (below) show the change in power during stimulus period from baseline in the slow gamma range during the respective protocols. (E) Time–frequency spectrogram for the meditators (left) and controls (right) for -0.5 to 1 second relative to the stimulus onset. The dashed lines show the slow gamma frequency range (24–34 Hz).

We next examined whether meditators have more stimulus-induced gamma than controls. First, we computed the PSDs for the occipital group during the spontaneous periods between the stimuli (-1 to 0 seconds; where 0 indicates stimulus onset) and found that PSDs during this epoch were similar to the EO1 condition for both G1 (n = 29) and G2 (n = 28) protocols (Fig. 3A; compare solid vs. dashed lines), with significantly higher broadband gamma in meditators than controls as in EO1 (see Supplementary Fig. S4 for statistical comparison). PSDs computed during the stimulus period (0.25 to 1.25 seconds after stimulus onset; Fig. 3B) revealed a prominent narrowband gamma bump, with a peak near 25 Hz (Fig. 3B), which was more prominent in meditators versus controls for both G1 and G2 (Supplementary Fig. S3B). This increase, however, is unsurprising since meditators had elevated power even during spontaneous periods (Fig. 3A).

To test whether the visual stimuli induced stronger gamma, we computed the change in power during the stimulus period compared with the baseline (Fig. 3C). The change in power plots also revealed the fast gamma between 40 and 70 Hz, which was not readily visible in the raw PSDs due to the line noise, as well as the suppression in alpha power around ~10 Hz due to stimulus onset. Interestingly, while meditators had similar changes as controls in alpha and fast gamma bands, there was a significantly higher slow gamma increase in meditators than controls for both G1 (t_(28)_ = 2.26, p = 0.032) and G2 (t_(27)_ = 2.98, p = 0.006) protocols (Fig. 3C), albeit the increase remained significant only for G2 after correcting for multiple comparisons (Table 1). Expectedly, this change in stimulus-induced slow gamma power was localized in the occipital regions (Fig. 3D). Time–frequency spectra (Fig. 3E) showed that the power in the slow gamma band increased over time, consistent with previous studies (Murty et al., 2018). Thus, even when the elevated spontaneous power is accounted for, meditators have stronger stimulus-induced slow gamma (but not fast gamma) than controls, even when they are not meditating.

We also performed a 2 × 2 mixed ANOVA to test whether pre-meditation baseline differences contributed to the observed group differences in stimulus-induced slow gamma (24–34 Hz) following meditation. Neither the main effect of Group (F_(1,26)_ = 2.786, p = 0.107, η² = 0.057) nor Protocol (F_(1,26)_ = 0.392, p = 0.537, η² = 0.000) reached significance and the analysis revealed no significant Group × Protocol interaction either (F_(1,26)_ = 3.544, p = 0.071, η² = 0.001), indicating that the magnitude of group differences did not change significantly from pre- to post-meditation. These results suggest that pre-meditation baselines did not drive the observed post-meditation group differences.

### 3.5 Stimulus-induced and stimulus-free gamma co-exist but are unrelated

**Fig. 4.**
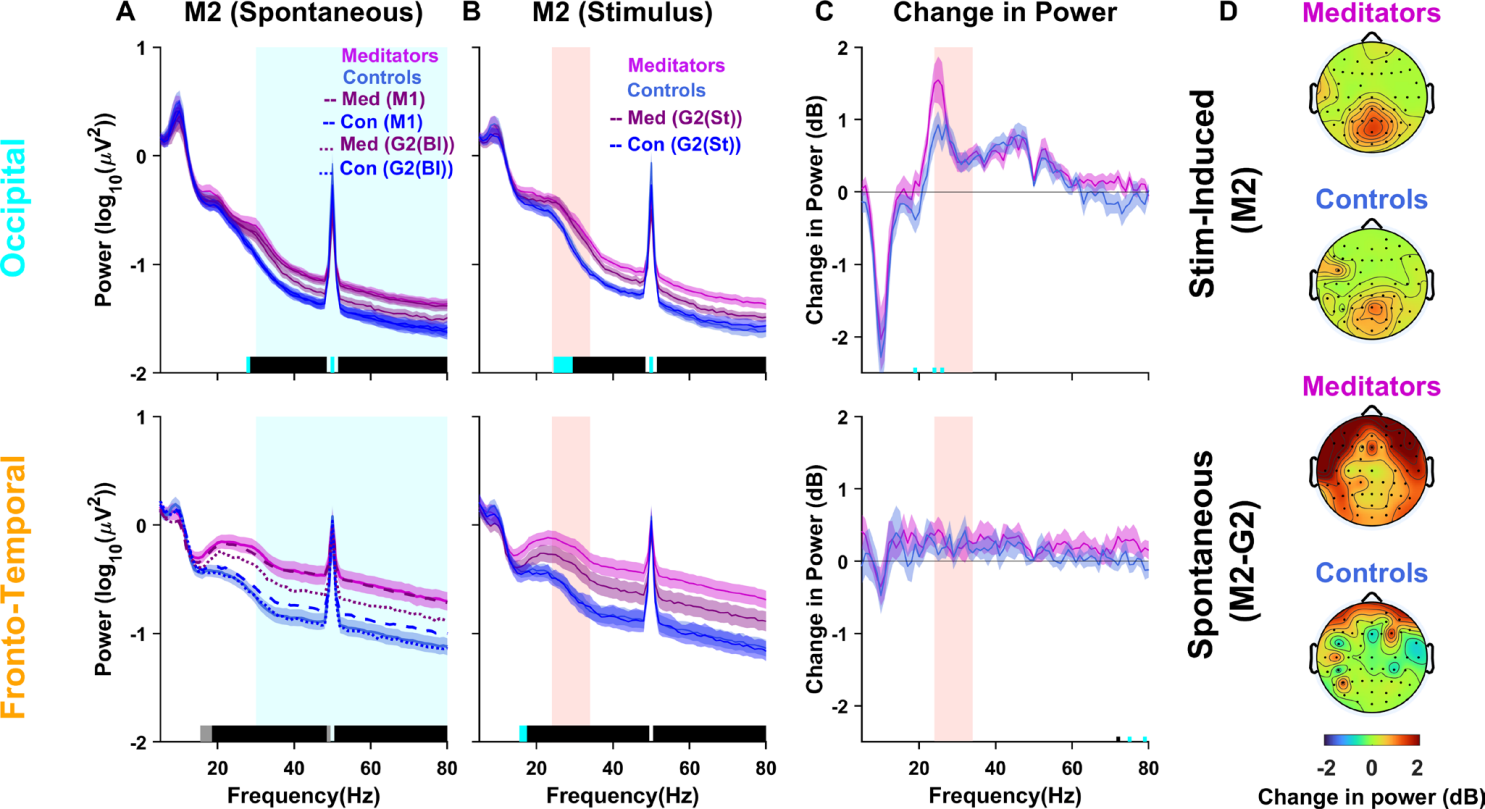
Stimulus-induced and stimulus-free gamma co-exist. (A) Average PSDs for the meditators (magenta) and controls (blue) for the M2 spontaneous (baseline) period for the occipital (top row; n = 29) and fronto-temporal (bottom row; n = 30) electrode groups (as shown in Fig. 2D top panel). Solid traces represent the mean and the shaded region around them indicates SEM across participants. Dashed and dotted lines show corresponding PSDs for the M1 and G2 baseline (Bl) periods. Horizontal bars at the abscissa represent the significance of differences in mean (gray: p < 0.05 and black: p < 0.01, paired t-test). (B) PSDs for the stimulus period (0.25 to 1.25 seconds) for the M2 protocol. Dashed lines show PSDs for the G2 protocol for the stimulus (St) period. (C) Change in power in dB during stimulus with respect to baseline for the M2 protocol. (D) The top two panels show the topoplots for change in power during stimulus with respect to baseline during M2 (n = 29), while the bottom panels show the topoplots for change in spontaneous power of M2 with respect to spontaneous power in G2 (n = 27).

Finally, we investigated the effect of meditation on stimulus-induced narrowband gamma (M2 protocol). First, we computed PSDs during spontaneous periods. If the participants were indeed able to meditate despite the continuous presentation of the stimuli, we expected these PSDs to be comparable with the PSDs obtained during M1 and further elevated compared with the spontaneous PSDs during non-meditative condition (e.g., EO2 or spontaneous condition in G2). We found both conditions to be true for meditators: spontaneous M2 PSDs (solid magenta lines in Figure 4A) were comparable with M1 PSDs (dashed magenta lines) and were elevated compared with the spontaneous G2 condition (dotted magenta lines; similar results were obtained if EO2 was used; n = 29, occipital; n = 30, fronto-temporal). For controls, the increase in broadband gamma power in the fronto-temporal region during M1 (dashed blue line compared with dotted blue line in the bottom row) was not observed during M2 (solid blue line compared with dotted blue line). This could potentially be due to more interference experienced by controls than meditators due to the stimuli.

Presentation of the stimulus produced stimulus-induced gamma in the occipital areas (Fig. 4B), which is better observed after comparing with the spontaneous period as done previously (Fig. 4C). We again observed a higher increase in slow gamma power in meditators compared with controls in the occipital area in the slow gamma range, even though the results did not reach significance when power was computed over the slow gamma range of 24–34 Hz used earlier (t_(28)_ = 1.34, p = 0.192; Fig. 4C; top row and Supplementary Fig. S5C), although it reached significance when slow-gamma was taken between 20 and 30 Hz (t_(28)_ = 2.1, p = 0.045). Note that in this case, the spontaneous PSD was already elevated due to meditation and, therefore, stimulus-induced gamma was computed with respect to this elevated PSD. Consequently, the increase in stimulus-induced gamma was somewhat less prominent than the increase observed without meditation (compare the scalp maps shown in the top two rows of Fig. 4D, which correspond to stimulus vs. baseline in M2, with the corresponding plots in G1 or G2 as shown in Fig. 3D). Also, note that the PSDs during stimulus periods of M2 were more elevated than the stimulus period of G2 (compare solid vs. dashed lines in Fig. 4B) for meditators but not controls, suggesting that meditation-induced stimulus-free (broadband elevation in power) and stimulus-induced gamma (narrowband increase in slow gamma as shown in Fig. 4C) co-exist.

To find the spatial localization of meditation-induced stimulus-free gamma, we computed the change in broadband power during spontaneous periods of M2 versus G2 (difference between the solid and dotted lines in Fig. 4A) and generated scalp maps (Fig. 4D, bottom two plots). The distribution of the meditation-induced broadband gamma was similar for meditators between M2 and M1 (compare Fig. 4D, third row vs. Fig. 2D, top row), but this increase was less prominent for controls during M2 (Fig. 4D, bottom row) than during M1 (Fig. 2D, second row). We tested whether participants who had a higher increase in meditation-induced gamma also had more stimulus-induced gamma. This was first tested by computing meditation-induced gamma during M1 (change in M1 vs. EO1 as shown in Fig. 2D) and stimulus-induced gamma during G1/G2 (change in stimulus vs. baseline as shown in Fig. 3D) and calculating the correlation. We found that the correlations were not significant between the two gamma signatures for any brain region for either meditator or control participants. For example, Supplementary Figure S6A shows the scatter plot for meditation-induced occipital gamma versus stimulus-induced occipital gamma (Pearson (r) and Spearman (ρ) correlations and the corresponding p-values are indicated in the plot); similar results were obtained when we compared gamma from fronto-temporal electrodes or compared meditation-induced fronto-temporal gamma with stimulus-induced occipital gamma (data not shown). Similarly, correlations between the change in power for stimulus-induced gamma during M2 (Fig. 4D, top plots) and meditation-induced gamma in M2 (Fig. 4D, bottom two plots) were not significant (Supplementary Fig. S6B). Hence, the two gamma signatures were distinct, potentially reflecting different mechanisms of generation.

#### 3.6 Source Localization

#### 3.6.1 Source localization of meditation-related broadband gamma (M1)

**Fig. 5.**
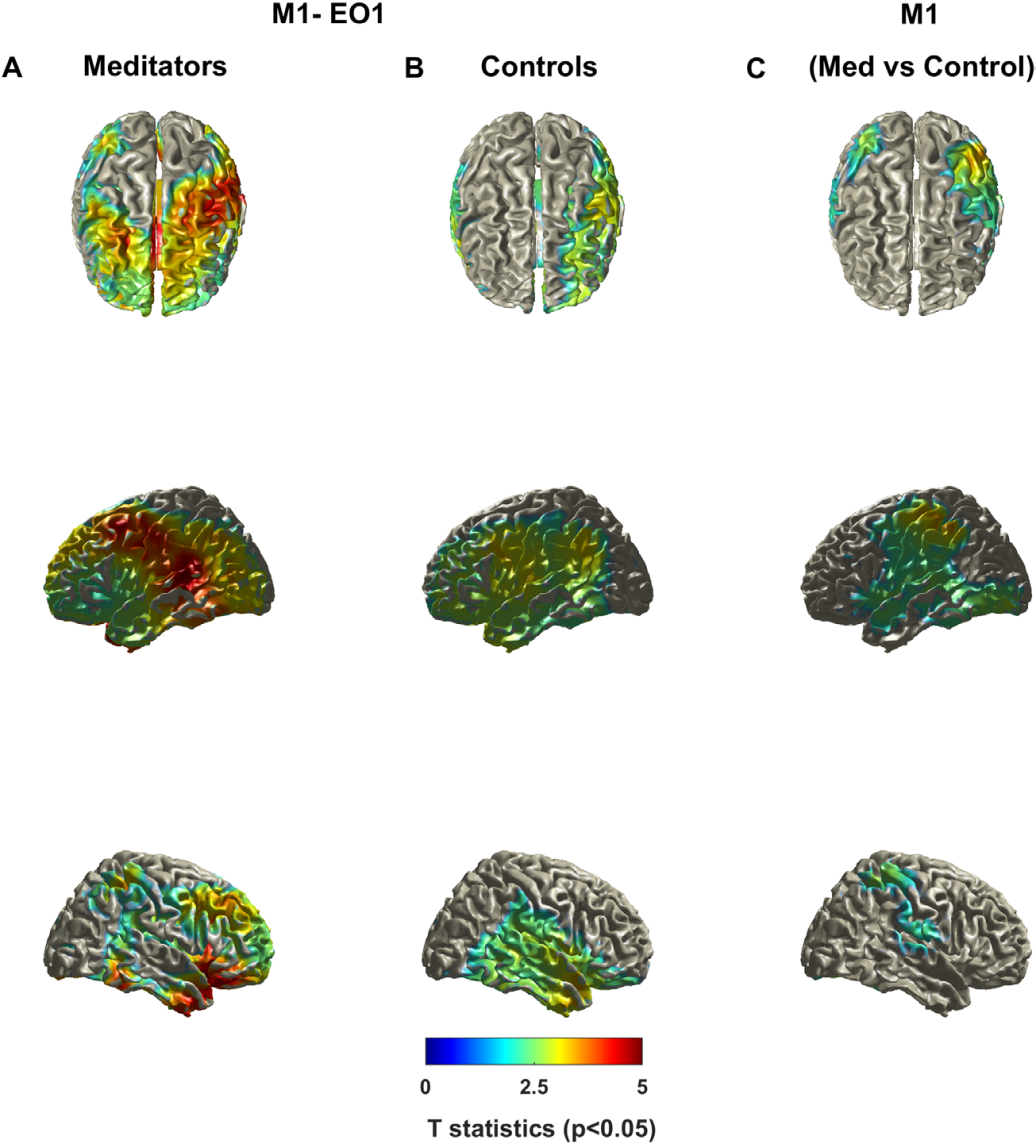
Cortical source t-statistic maps of broadband gamma activity during meditation (M1). (A) Surface-rendered t-statistic maps for the meditator group, showing significant cortical sources during meditation (M1) compared with eyes-open rest (EO1), displayed in superior (top), left lateral (middle), and right lateral (bottom) views. (B) Corresponding maps for the control group for the same contrast (M1 vs. EO1). (C) Group-level comparison between meditators and controls during meditation (M1), showing regions with significantly stronger broadband gamma activity in meditators. All maps are thresholded at p < 0.05 and color coded for t-values (blue–red scale).

To identify the cortical sources underlying enhanced gamma activity observed in meditators, we performed source localization analysis on the broadband gamma power during meditation (M1). Figure 5A shows the surface-rendered t-statistic maps for meditators during M1 compared with their eyes-open rest condition (EO1). The analysis showed widespread cortical gamma sources across multiple brain regions, with the strongest activity concentrated in frontal, parietal, and temporal regions. In contrast, the control group showed markedly weaker gamma sources during the same meditation period (M1 vs. EO1; Fig. 5B). Direct group comparison between meditators and controls during meditation (Fig. 5C) confirmed that meditators exhibited significantly stronger broadband gamma activity across distributed cortical regions, with the most prominent differences observed bilaterally in posterior parietal and lateral temporal cortices.

#### 3.6.2 Source localization of stimulus-induced slow gamma (G1, G2)

**Fig. 6.**
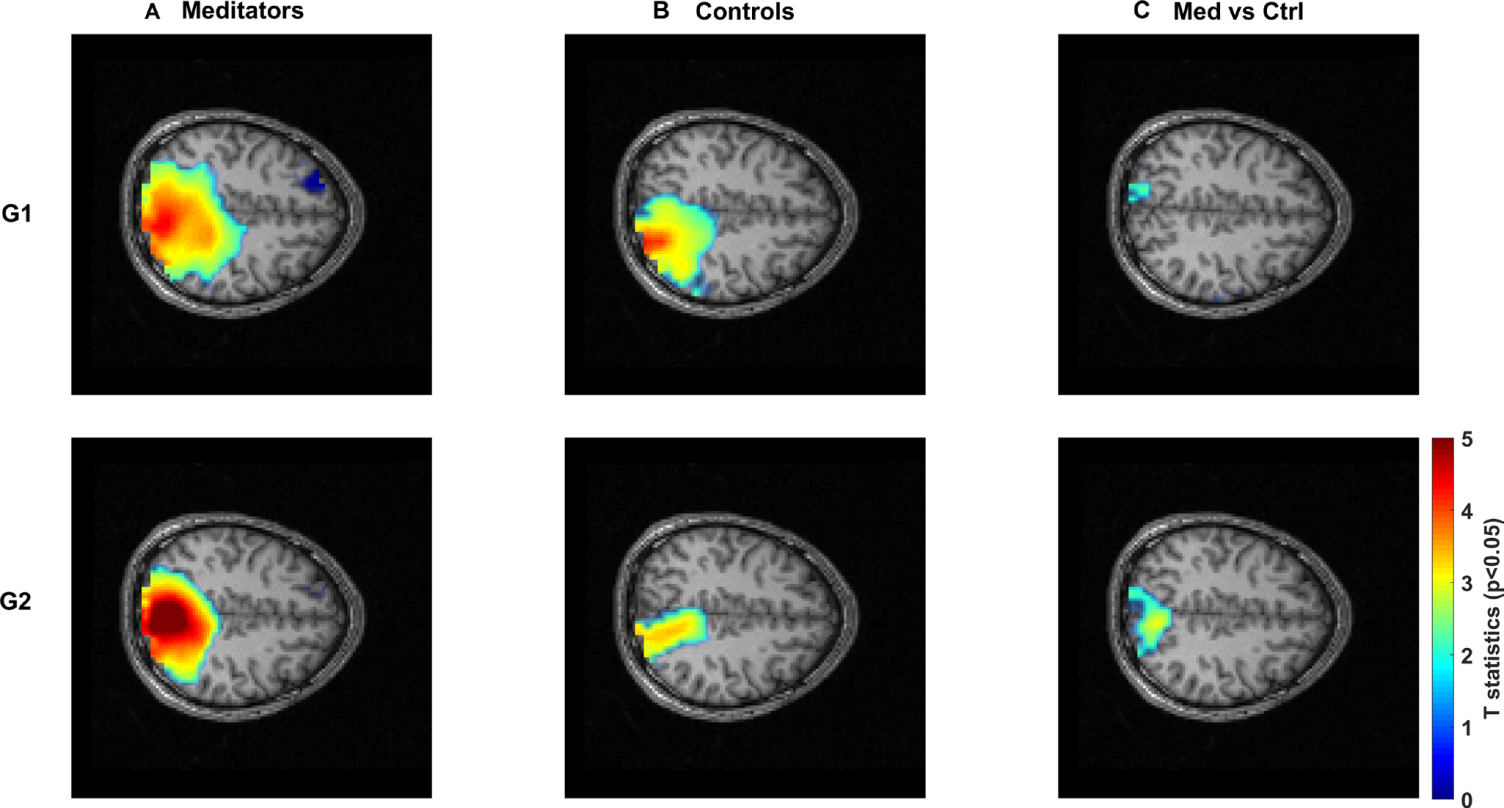
Cortical sources of stimulus-induced slow-gamma (G1, G2). Axial t-statistic maps are shown for the first (G1, top row) and second (G2, bottom row) gamma protocols. Columns: (A) meditators, stimulus versus pre-stimulus baseline; (B) controls, stimulus versus pre-stimulus baseline; (C) group comparison during the stimulus epoch (meditators > controls). Maps are thresholded at p < 0.05, overlaid on the standard MNI template; the color bar denotes t-values (blue–red). In both sessions, sources localized predominantly to occipital cortex, with meditators exhibiting stronger activation.

We next examined the cortical distribution of stimulus-induced slow-gamma activity (G1 and G2) to determine whether the enhanced gamma responses in meditators originated from similar or distinct cortical networks compared with spontaneous gamma during meditation. Figure 6 shows axial t-statistic maps for both gamma protocols overlaid on the standard MNI template.

For the first gamma protocol (G1, top row), meditators showed prominent cortical sources in occipital cortex during the stimulus period compared with pre-stimulus baseline (Fig. 6A). Controls similarly exhibited occipital gamma sources during G1, though with lower amplitude and more restricted spatial extent (Fig. 6B). Group comparison revealed that meditators had significantly stronger activation in occipital regions (Fig. 6C). The second gamma protocol (G2, bottom row) showed a similar spatial pattern, with both groups showing predominantly occipital sources (Fig. 6A, B). However, meditators again demonstrated stronger and more spatially extensive activation that extended into parieto-occipital regions (Fig. 6C). Notably, across both G1 and G2 sessions, the stimulus-induced gamma sources localized predominantly to occipital cortex in both groups.

### 3.7 Relationships between self-report scores and EEG (power and slope)

We also recorded personality traits and mystical experience using a self-reported standard set of questionnaires (see Section 2 for details). Meditators had significantly higher mindfulness and mystical experience scores and lower stress scores than controls (Supplementary Fig. S7; p-values indicated in the plots). However, none of these scores correlated with gamma power (Supplementary Fig. S7; correlation between Mindfulness Score and gamma was significant for Meditators with p = 0.04 but that was due to one outlier point since removing that point or performing a Spearman rank correlation yielded non-significant correlation). We also tested the correlation between the PSD slopes and test scores (Supplementary Fig. S8). We did not find any consistent pattern, except a weak anti-correlation between slopes and stress scores in meditators during EC1.

Finally, we also tested whether meditation-induced or stimulus-induced gamma depended on the number of hours of meditative practice but found no significant correlation (data not shown). We note that our study was not designed to test for this, since we did not explicitly recruit meditators with varying experience. Further, stimulus-induced gamma, although consistent across multiple recordings for a given individual, varies considerably across participants (Kumar et al., 2022). In our data also, we found that the time–frequency plots (as shown in Fig. 3E) for G1, G2, and M2 were very similar for individual participants, but there was considerable variability across participants (data not shown).

## 4 Discussion

In the present study, we asked three questions: first, whether long-term meditators have more stimulus-induced gamma. Second, whether the stimulus-induced gamma co-exists with the previously shown stimulus-free endogenous gamma. Third, how meditation changes the slope of the power spectral density. We found that meditators have stronger stimulus-free and stimulus-induced gamma than age- and gender-matched control participants. These gamma signatures are prominent in fronto-temporal and parieto-occipital areas and can co-exist when stimuli are presented while they meditate but are unrelated. Further, PSD slopes are steeper for meditators than for controls. We also obtained self-reported levels of mindfulness, happiness, stress, and mystic experience from our participants but found no correlation with gamma power or PSD slopes, as discussed later.

### 4.1 Effect of meditation on gamma

Long-term meditators had more broadband power in the gamma frequency range (>30 Hz) during stimulus-free meditation, consistent with several previous reports (Braboszcz et al., 2017; Cahn et al., 2010; Lutz et al., 2004). Importantly, we observed that long-term meditators had elevated stimulus-induced “narrowband” slow-gamma, but not fast-gamma power even before they meditated, which hints toward long-term changes in the brain, also known as a trait effect (Cahn & Polich, 2013). However, we did not observe any significant changes in other frequency bands such as delta (1–3 Hz) or alpha (8–13 Hz), as shown previously (Cahn et al., 2013; Lagopoulos et al., 2009; Stapleton et al., 2020) for some other meditation practices. This might be because previous studies have mainly studied eyes-closed meditation practices in which alpha rhythm is prominent, while we studied meditation during eyes-open states where low-frequency rhythms were weaker, to begin with. Further, it has been previously shown that only some forms of meditation increase alpha power. For example, meditators following Himalayan yoga traditional or Isha shoonya yoga had comparable alpha as controls, but the Vipassana group had stronger alpha (see figure 3 of Braboszcz et al., 2017).

### 4.2 Different origins of gamma power

Interestingly, we found that broadband stimulus-free gamma and stimulus-induced narrowband slow gamma can co-exist but are unrelated. It indicates that these two gamma signatures may have different origins. Slow gamma during meditation could be generated due to the dendrite-targeting inhibitory somatostatin network, although this has been shown mainly in rodents (Chen et al., 2017; Veit et al., 2017). It is coherent over larger distances as compared with fast gamma when recorded using both microelectrode arrays (Murty et al., 2018) and EEG (Kumar & Ray, 2023), and, therefore, could be thought of as a “global” gamma compared with a more local fast gamma thought to be generated due to the soma-targeting parvalbumin-positive interneurons (Bartos et al., 2007; Buzsáki & Wang, 2012). In comparison, the broadband increase in power, which has been observed in many meditation studies (Braboszcz et al., 2017; Cahn et al., 2010; Lutz et al., 2004), has been associated with increased spiking activity in microelectrode recordings (Ray & Maunsell, 2011) and could reflect enhanced synchrony in macro-signals (Ray et al., 2008).

Although we use the term *stimulus-free gamma* to describe the broadband elevation of high-frequency power observed during meditation, we acknowledge that this activity lacks a distinct oscillatory peak in the power spectrum and, therefore, does not constitute a “gamma rhythm” in the strict spectral sense. Our terminology follows prior EEG studies of long-term meditation practitioners (Braboszcz et al., 2017; Cahn et al., 2013; Lutz et al., 2004), which reported broadband increases above ~25–30 Hz under similar conditions and we adopted the same convention for comparability. Thus, the term *gamma* here is used in a descriptive sense, referring to sustained high-frequency neural activity rather than to a narrowband oscillation with a well-defined peak. This distinction is conceptually important, as it underscores that the broadband, stimulus-free enhancement and the narrowband, stimulus-induced gamma likely reflect distinct neurophysiological generators and functional processes.

Beyond gamma oscillations, other neural signatures have been linked to meditation practice. In particular, heartbeat-evoked potentials (HEPs)—EEG potentials time locked to the cardiac R-peak—have emerged as a complementary biomarker. HEPs index cortical processing of cardiac signals and are modulated by interoceptive attention and changes in bodily self-awareness during meditation (Park et al., 2016; Yang et al., 2025). Thus, while endogenous gamma may reflect local cortical activation dynamics, HEPs appear to capture alterations in interoceptive processing and the sense of bodily self. Future studies analyzing both measures together could clarify whether these biomarkers are independently modulated by meditation practice or share common underlying mechanisms.

#### 4.2.1 Source distribution of meditation-induced and stimulus-induced gamma

Meditation-induced broadband gamma showed widespread distribution across frontal, parietal, and temporal cortices, consistent with previous MEG studies demonstrating long-range cortical synchronization in experienced meditators (Lutz et al., 2004, 2008, 2009). This fronto-temporo-parietal distribution aligns with the cognitive demands of Rajyoga practice, which requires transitioning from body consciousness to sustained internal awareness directed toward an imagined higher being (Nair et al., 2017; Sharma et al., 2023). Frontal activation likely reflects sustained cognitive control and meta-awareness essential for maintaining meditative focus (Cahn & Polich, 2006; Hasenkamp et al., 2012; Tang et al., 2015), while parietal engagement supports attentional demands and temporal involvement relates to altered self-referential processing. This pattern is consistent with meditation studies showing default mode network deactivation coupled with frontoparietal control region engagement during focused attention states (Brewer et al., 2011; M. D. Fox et al., 2005). The distributed gamma activity may facilitate communication between spatially distant cortical areas, supporting integration of attention, awareness, and interoceptive processing characteristic of meditative states (Singer, 1999; Varela et al., 2001).

In contrast, stimulus-induced gamma was localized predominantly to occipital cortex, consistent with its well-established role in visual processing (Biswas et al., 2025; Gray et al., 1989; Murty et al., 2018). Critically, this enhanced stimulus-induced gamma persisted during passive visual stimulation outside formal meditation, demonstrating trait-level changes in cortical function rather than purely state-dependent effects (K. C. R. Fox et al., 2014). The striking difference in spatial distribution between stimulus-induced gamma (predominantly occipital) and meditation-induced gamma (widespread across frontal-parietal-temporal networks) indicates fundamentally distinct neural mechanisms. Stimulus-induced gamma could reflect bottom-up sensory processing amplified by enhanced cortical inhibition in visual areas, whereas meditation-induced gamma could represent top–down cognitive control and large-scale network integration across distributed cortical regions (Engel et al., 2001; von Stein & Sarnthein, 2000).

### 4.3 Meditation steepens the slope of the power spectral density

The slope of the PSD reflects the aperiodic “1/f” component and has been associated with many different processes, such as self-organized criticality (He, 2014), excitation–inhibition balance (Freeman & Zhai, 2009; Gao et al., 2017), neural noise (Voytek et al., 2015), and temporal dynamics of synaptic processes (Milstein et al., 2009). A recent study showed a steepening slope during meditation versus non-meditative conditions for meditators (Rodriguez-Larios et al., 2021). We also observed a similar increase in slopes for both meditators and controls when they meditated, but the increase was not significant. The difference could be due to differences in meditation techniques (closed-eye mindfulness, Zen, and Vipassana meditation vs. open-eye BK Rajyoga meditation) or the use of a different frequency range (2–30 Hz vs. 104–190 Hz).

We used a higher frequency range to avoid modulation of slopes due to oscillatory activity at lower frequencies and because we have previously shown that the flattening of PSDs with aging can be observed at higher frequencies as well (Aggarwal & Ray, 2023). The high-frequency activity above 100 Hz could reflect neural processes that have timescales less than 10 ms, for example, spike propagation (Shepherd, 2004) and synaptic neurotransmitter diffusion (Sabatini & Regehr, 1996), although in EEG, further experiments are needed to identify these sources. The high-frequency range (>100 Hz) in EEG is susceptible to noise and artifacts, which could have electromyographic (EMG) origins or could be due to amplifier or instrument noise (Muthukumaraswamy, 2013; Whitham et al., 2007). Indeed, we found that PSD slopes were generally steeper during EO1 versus EC1 (compare the slopes in Fig. 2B with 2A), which could be due to some increase in EMG-related power between 50 and 150 Hz (which can be observed by comparing the PSDs for EO1 vs. EC1). However, it is unlikely that the steeper PSD slopes for meditators versus controls is only due to EMG-related artifacts, since differences in PSD slopes between meditators and controls were observed even for the eyes-closed condition.

### 4.4 Potential effect of attention, eye movements, and electrode impedance on gamma

Besides meditation, factors such as eye movements (Yuval-Greenberg et al., 2008) and attention (Fries et al., 2001) are also known to affect gamma power. So, could our results be explained based on differences in potential attentional load or eye movements between meditators versus controls (e.g., if the meditators paid more attention to the visual stimuli than controls)? Although these possibilities cannot be completely ruled out, they are unlikely to play a major role here because meditation mainly had an effect on slow-gamma but not alpha or fast-gamma bands. In EEG, the main effect of attention is to reduce alpha band power, while there is some enhancement of gamma (Das et al., 2024; Foxe & Snyder, 2011). Therefore, differences in attentional load should have been reflected in the alpha power, which we did not observe. Similarly, micro-saccades increase power over a broad frequency range in the gamma range (Yuval-Greenberg et al., 2008), and, therefore, the fast gamma power should have been different if there was a difference in the way meditators and controls maintained fixation during stimulus presentation. Longer saccades are anyway ruled out in our data since we only analyzed data for trials where participants kept their gaze within 2.5 degrees of the fixation point (see Section 2 for details). Finally, we tested whether electrode impedance has any effect on gamma power or the PSD slope since impedance could affect EEG data quality (Dimakopoulos et al., 2023; Kappenman & Luck, 2010). However, we could not find any correlation between the electrode impedance and gamma power or PSD slope (data not shown).

To address multiple comparison concerns for the test statistics given in Table 1 and Supplementary Tables S1–S3, we applied False Discovery Rate (FDR) correction (see Section 2 for details). This was done at two levels: across protocols for each frequency band and across individual electrode groups. This approach balances sensitivity and specificity while avoiding excessive conservatism of the Bonferroni correction (e.g., α ≈ 0.00046 for 108 tests) and effectively controlling false positives (Benjamini & Hochberg, 1995; Verhoeven et al., 2005). Importantly, core findings, such as group differences in the occipital cluster in protocol G2, remained significant after correction.

We did not have any additional cognitive task (such as an attention task) because the experimental session was long and tedious for the participants, with two separate meditation sessions. Therefore, potential relationship between gamma and cognitive performance could not be addressed here. Instead, our focus was to simply characterize stimulus-induced gamma power in meditators versus controls since these oscillations are linked to inhibitory circuitry. Although we did not perform any additional cognitive task, we used several questionnaires to determine mindfulness, happiness, stress, and mystic experience levels of our participants. While these scores varied in expected ways between meditators and controls, none of these scores correlated with gamma changes. This is not surprising, since we have previously shown that gamma power varies considerably across individuals, even when they are deemed healthy based on multiple questionnaires (Kumar & Ray, 2022; see Figs.1 and 2 for gamma response of 20 healthy male and female participants). Differences across individuals could simply be due to external factors such as shape of the skull, resistance of the skin, position of the gyri and sulci, etc., which are unrelated to cognition. However, although the gamma response varied considerably across individuals, it remained remarkably consistent across multiple recordings within an individual, even when the recordings were separated by 1 year (Kumar & Ray, 2022).

Overall, attention or other cognitive factors that modulate alpha/fast gamma bands as well are unlikely to completely explain our results. Instead, the selective increase in slow-gamma power as well as steeper PSD slopes in meditators both provide indications of a stronger long-range inhibitory circuit. As slow-gamma (but not alpha or fast gamma) connectivity is selectively impaired in MCI patients (Kumar & Ray, 2023), the enhanced slow-gamma activity in meditators suggests that meditation could help mitigate age or mental disorder-related neural changes. However, establishing causality will require future longitudinal and interventional studies.

## Supplementary Material

Supplementary Material

## Data Availability

All spectral analyses were performed using the Chronux toolbox (version 2.10), available at https://chronux.org. Slopes were obtained using MATLAB wrapper for FOOOF (github.com/fooof-tools/fooof_mat). Codes to view the analyzed power data are available on GitHub at https://github.com/supratimray/ProjectDhyaanBK1Programs.
