# Supplementary material for: Simultaneous enhancement of stimulus-induced and stimulus-free gamma in open-eye meditators
Source: Imaging Neurosci (Camb). 2026 Feb 25;4:IMAG.a.1145. doi: 10.1162/IMAG.a.1145 (PMC12938738; doi:10.1162/IMAG.a.1145)
Supplement: Supplementary Material [file IMAG.a.1145_supp.pdf]

Supplementary Materials

Supplementary Table 1. P-values and statistics of the independent-samples t-tests performed between unpaired meditators and controls for different frequency bands for all the protocols for occipital and fronto-temporal electrodes, following the same format as Table 1. Group-specific sample sizes (**n1**: meditators; **n2**: controls) are displayed in parentheses for each protocol. Degrees of freedom (**df**) were calculated as **n1 + n2 − 2** for unpaired t-tests. See Table 1 legend for details.

	**Band**		**Protocols**								
**Occipital electrode group**			**EO1 (34,34)**	**EC1 (35,35)**	**G1 (35, 34)**	**M1 (33,34)**	**G2 (34,33)**	**EO2 (35,28)**	**EC2 (34,36)**	**M2_bl (35, 35)**	**M2_st (35,35)**
	Delta (1-3 Hz)	t =	1.48	1.71	-0.51	0.66	0.45	-0.05	0.21	-0.9	-0.93
		p =	0.144	0.091	0.615	0.512	0.653	0.96	0.836	0.371	0.357
	Theta (4-7 Hz)	t =	1.62	1.75	0.56	0.72	-0.74	0.18	1.49	0.28	0.65
		p =	0.109	0.085	0.577	0.476	0.464	0.86	0.141	0.779	0.52
	Alpha (8-12 Hz)	t =	0.82	0.48	0.34	-0.15	0.79	1.08	0.3	0.49	0.77
		p =	0.418	0.63	0.733	0.883	0.431	0.284	0.763	0.624	0.444
	Beta (13-23 Hz)	t =	1.87	1.28	1.94	1.11	1.03	1.81	1.61	1.37	2.03
		p =	0.067	0.206	0.057	0.27	0.309	0.074	0.112	0.176	†0.046
	Slow gamma (24-34 Hz)	t =	2.8	2.61	1.4	2.38	2.7	2.3	3.27	3.23	3.57
		p =	**†0.007**	**†0.011**	0.1668	†0.02	**†0.009**	†0.025	**†0.002**	**†0.002**	**†7×10-4**
	Broadband gamma (30-80 Hz)	t =	2.82	1.8	0.23	3.57	1.75	1.66	2.8	4.56	4.65
		p =	**†0.006**	0.077	0.821	**†7×10-4**	0.085	0.102	**†0.007**	**†2×10-5**	**†2×10-5**
**Fronto-temporal electrode group**			**EO1 (34,34)**	**EC1 (35,36)**	**G1 (35,34)**	**M1 (33,34)**	**G2 (34,34)**	**EO2 (35,29)**	**EC2 (35,36)**	**M2_bl (35,36)**	**M2_st (35,36)**
	Delta (1-3 Hz)	t =	1.5	0.98	1.19	-0.03	-0.78	-0.98	-0.07	1.41	1.48
		p =	0.139	0.33	0.238	0.977	0.437	0.33	0.944	0.163	0.142
	Theta (4-7 Hz)	t =	1.42	1.09	1	-0.39	-0.4	-1.83	0.27	1.24	1.38
		p =	0.16	0.28	0.321	0.7	0.693	0.072	0.785	0.22	0.172
	Alpha (8-12 Hz)	t =	0.59	0.13	1.53	-0.29	-0.46	-0.78	0.57	0.72	0.72
		p =	0.558	0.896	0.132	0.775	0.65	0.44	0.569	0.472	0.477
	Beta (13-23 Hz)	t =	2.4	2	1.18	2.08	-0.85	0.04	2.04	3.44	3.29
		p =	*0.019	*0.049	0.243	3.59	0.396	0.967	*0.045	**†0.001**	**†0.002**
	Slow gamma (24-34 Hz)	t =	3.6	3.7	0.34	3.59	-0.43	1.43	3.22	4.78	4.83
		p =	**†0.001**	**†4×10-04**	0.733	**†6×10-4**	0.667	0.157	**†0.002**	**†1×10-5**	**†1×10-5**
	Broadband gamma (30-80 Hz)	t =	3.37	3.42	0.83	3.62	-1.11	1.49	2.46	4.69	4.77
		p =	**†0.001**	**†0.001**	0.409	**†6×10-4**	0.27	0.141	†0.016	**†1×10-5**	**†1×10-5**

Supplementary Table 2. P-values and statistics of the Wilcoxon signed-rank tests performed between paired meditators and controls for different frequency bands for all the protocols for occipital and fronto-temporal electrodes, following the same format as Table 1. Sample sizes (n pairs) are displayed in parentheses for each protocol. See Table 1 legend for details.

	Band		Protocols								
**Occipital Group**			**EO1 (29)**	**EC1 (29)**	**G1 (29)**	**M1 (29)**	**G2 (28)**	**EO2 (25)**	**EC2 (29)**	**M2_bl (29)**	**M2_st (30)**
	Delta (1-3 Hz)	W =	275	264	221	249	262	181	220	151	154
		p =	0.214	0.315	0.940	0.496	0.079	0.619	0.957	0.150	0.170
	Theta (4-7 Hz)	W =	290	261	244	249	173	168	252	210	222
		p =	0.117	0.347	0.567	0.496	0.701	0.882	0.456	0.871	0.922
	Alpha (8-12 Hz)	W =	216	225	239	190	212	180	214	204	205
		p =	0.974	0.871	0.642	0.552	0.581	0.638	0.940	0.770	0.787
	Beta (13-23 Hz)	W =	256	227	300	225	219	197	236	243	266
		p =	0.405	0.837	0.074	0.871	0.471	0.353	0.689	0.581	0.294
	Slow gamma (24-34 Hz)	W =	302	304	313	288	287	199	323	322	342
		p =	0.068	0.061	*0.039	0.127	*0.019	0.326	*0.023	*0.024	*0.007
	Broadband gamma (30-80 Hz)	W =	306	308	206	350	254	174	349	384	392
		p =	0.056	0.050	0.804	†0.004	0.118	0.757	†0.004	**†3.2×10⁻⁴**	**†1.6×10⁻⁴**
**Fronto-temporal Electrode Group**			**EO1 (29)**	**EC1 (30)**	**G1 (29)**	**M1 (29)**	**G2 (29)**	**EO2 (26)**	**EC2 (30)**	**M2_bl (30)**	**M2_st (30)**
	Delta (1-3 Hz)	W =	288	268	283	231	217	143	238	278	284
		p =	0.127	0.465	0.157	0.770	0.750	0.409	0.910	0.349	0.289
	Theta (4-7 Hz)	W =	266	246	252	205	217	96	225	230	244
		p =	0.294	0.781	0.456	0.787	0.750	*0.043	0.877	0.959	0.813
	Alpha (8-12 Hz)	W =	197	199	293	190	221	137	220	208	200
		p =	0.658	0.491	0.103	0.552	0.682	0.328	0.797	0.614	0.504
	Beta (13-23 Hz)	W =	300	303	280	316	207	172	280	349	355
		p =	0.074	0.147	0.177	*0.033	0.927	0.929	0.329	*0.017	**0.012**
	Slow gamma (24-34 Hz)	W =	348	400	230	344	254	220	363	397	402
		p =	**†0.005**	**†5.7×10⁻⁴**	0.787	**†0.006**	0.246	0.258	**†0.007**	**†7.2×10⁻⁴**	**†4.9×10⁻⁴**
	Broadband gamma (30-80 Hz)	W =	345	413	241	343	198	217	363	408	413
		p =	**†0.006**	**†2.1×10⁻⁴**	0.611	**†0.007**	0.909	0.292	**†0.007**	**†3.1×10⁻⁴**	**†2.1×10⁻⁴**

Supplementary Table 3. P-values and statistics of the Mann-Whitney U tests performed between unpaired meditators and controls for different frequency bands for all the protocols for occipital and fronto-temporal electrodes, following the same format as Table 1. Group-specific sample sizes (**n1**: meditators; **n2**: controls) are displayed in parentheses for each protocol. See Table 1 legend for details.

	Band		Protocols								
**Occipital Group**			**EO1 (34,34)**	**EC1 (35,35)**	**G1 (35, 34)**	**M1 (33,34)**	**G2 (34,33)**	**EO2 (35,28)**	**EC2 (34,36)**	**M2_bl (35, 35)**	**M2_st (35,35)**
	Delta (1-3 Hz)	U =	1234	1367	1253	1165	1278	1136	1211	1191	1183
		p =	0.458	0.145	0.741	0.594	0.128	0.830	0.967	0.549	0.488
	Theta (4-7 Hz)	U =	1299	1395	1251	1200	1119	1159	1336	1269	1307
		p =	0.124	0.074	0.760	0.331	0.647	0.594	0.131	0.760	0.452
	Alpha (8-12 Hz)	U =	1210	1281	1231	1087	1207	1181	1223	1258	1296
		p =	0.654	0.655	0.947	0.665	0.527	0.403	0.855	0.860	0.534
	Beta (13-23 Hz)	U =	1319	1336	1399	1201	1224	1238	1328	1362	1400
		p =	0.074	0.275	*0.037	0.325	0.397	0.104	0.157	0.162	0.065
	Slow gamma (24-34 Hz)	U =	1388	1422	1312	1275	1320	1259	1439	1472	1496
		p =	†0.009	*0.036	0.299	0.056	*0.040	0.055	†0.007	†0.007	**†0.003**
	Broadband gamma (30-80 Hz)	U =	1363	1357	1188	1392	1273	1230	1397	1583	1589
		p =	†0.020	0.181	0.661	**†7.3×10⁻⁴**	0.144	0.130	†0.026	**†6.5×10⁻⁵**	**†4.8×10⁻⁵**
**Fronto-temporal Electrode Group**			**EO1 (34,34)**	**EC1 (35,36)**	**G1 (35,34)**	**M1 (33,34)**	**G2 (34,34)**	**EO2 (35,29)**	**EC2 (35,36)**	**M2_bl (35,36)**	**M2_st (35,36)**
	Delta (1-3 Hz)	U =	1267	1336	1336	1133	1176	1110	1268	1403	1409
		p =	0.251	0.385	0.185	0.895	0.976	0.716	0.931	0.101	0.088
	Theta (4-7 Hz)	U =	1302	1328	1243	1140	1119	1067	1343	1408	1405
		p =	0.115	0.438	0.834	0.826	0.512	0.345	0.343	0.090	0.097
	Alpha (8-12 Hz)	U =	1217	1245	1341	1134	1165	1108	1324	1320	1329
		p =	0.594	0.868	0.166	0.885	0.927	0.696	0.465	0.494	0.431
	Beta (13-23 Hz)	U =	1368	1434	1341	1327	1135	1159	1464	1547	1543
		p =	†0.017	*0.046	0.166	**†0.010**	0.646	0.777	†0.019	**†9.8×10⁻⁴**	**†0.001**
	Slow gamma (24-34 Hz)	U =	1442	1542	1211	1418	1224	1241	1510	1631	1624
		p =	**†9.9×10⁻⁴**	**†0.001**	0.871	**†2.1×10⁻⁴**	0.536	0.165	**†0.004**	**†2×10⁻⁵**	**†2.9×10⁻⁵**
	Broadband gamma (30-80 Hz)	U =	1409	1526	1212	1415	1140	1241	1463	1616	1624
		p =	**†0.004**	**†0.002**	0.881	**†2.4×10⁻⁴**	0.690	0.165	†0.020	**†4.3×10⁻⁵**	**†2.9×10⁻⁵**

Supplementary figures

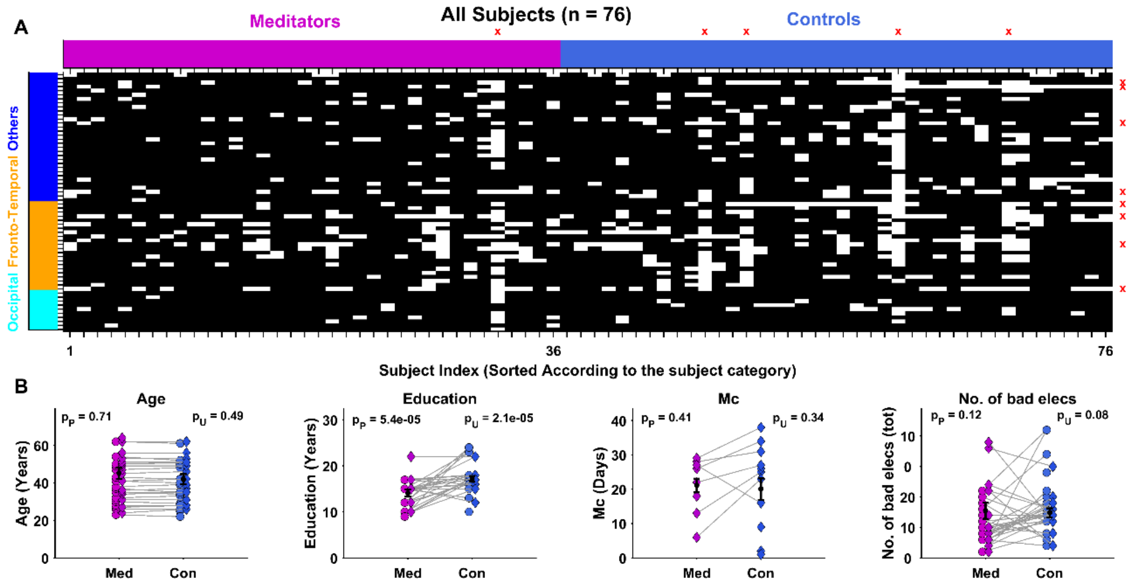

Supplementary Figure 1. Bad Electrodes and demographic distribution of Participants (A) Heat map showing the status of 64 electrodes across all 76 participants. The black and white colors indicate good and bad electrodes, respectively. Participants are shown on the x-axis after grouping them as meditators or controls. The participants with more than 40 bad electrodes are marked as red 'x' on the top abscissa and deemed bad (1 meditator and 4 controls). The electrodes are shown on the y-axis after arranging them in the order of occipital, fronto-temporal and remaining ("others") electrode groups. The electrodes that were bad in more than 35% of the participants in either meditator or control group are marked as red 'x' on the left ordinate and were rejected (8 bad electrodes). **(B)** The comparison of demographic details: age, education level, days from the first day of the last menstrual cycle for non-menopausal females and the number of bad electrodes between meditators and controls for all 71 good participants and 30 matched pairs. The circles and diamonds represent male and female participants, respectively. The pairs are indicated by grey lines joining the paired participants. Means are compared using both paired and unpaired independent-samples t-test, for which the p values are indicated on the top as p_P and p_U respectively.

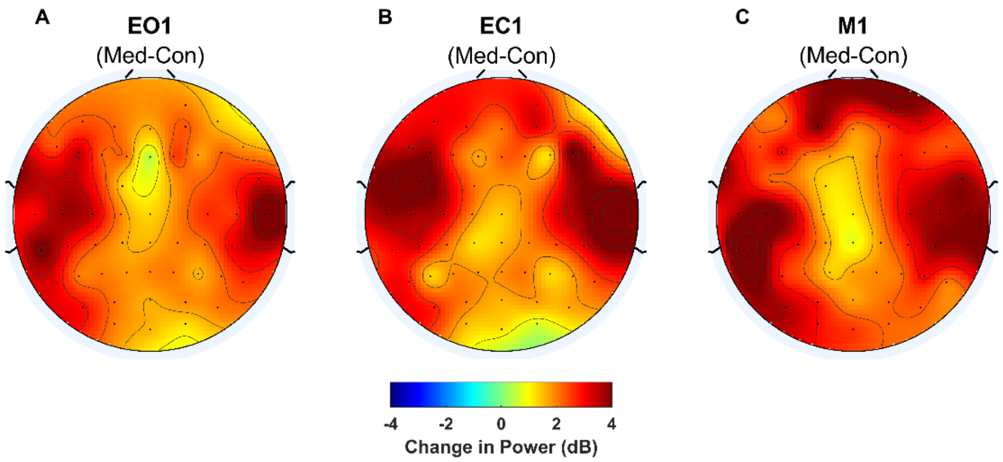

Supplementary Figure 2. Scalp maps showing the change in power (in decibels) in the broadband gamma frequency range (30-80 Hz) for meditators with respect to controls in **(A)** EO1, **(B)** EC1 and **(C)** M1. Meditators had more broadband gamma power than controls in all brain regions.

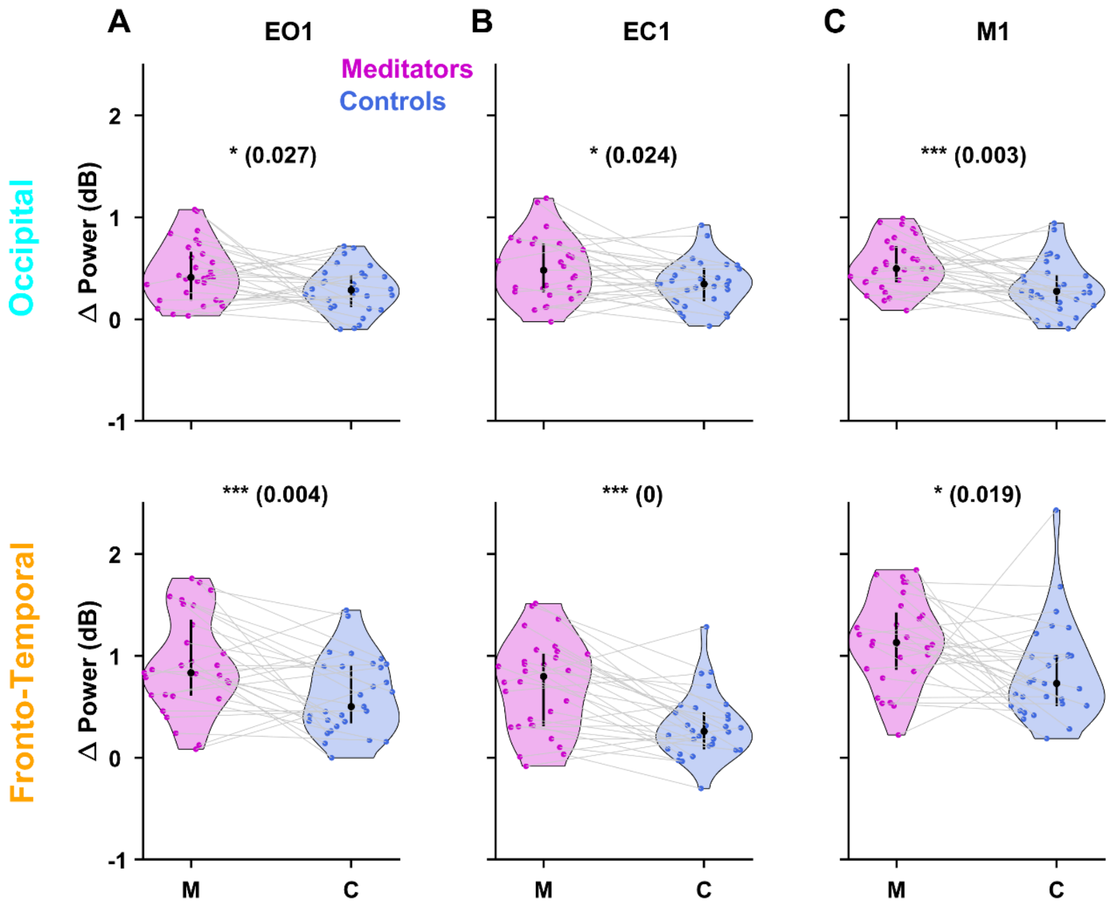

Supplementary Figure 3. Meditators show higher broadband gamma power across eyes-open, eyes-closed, and meditation conditions. Violin plots showing broadband gamma power (30–80 Hz) in occipital (top row, cyan labels) and fronto-temporal (bottom row, orange labels) electrode groups for meditators (magenta) and controls (blue) during (**A**) eyes-open rest (EO1), (**B**) eyes-closed rest (EC1), and (**C**) open-eye meditation (M1). Each dot represents an individual participant, with grey lines connecting age- and gender-matched pairs. Meditators exhibited significantly higher broadband gamma power than controls across all three conditions in both occipital and fronto-temporal regions, with effects strongest in the latter. Significance levels correspond to paired t-tests (* $p < 0.05$; ** $p < 0.01$; *** $p < 0.005$); exact p-values are shown in parentheses. Error bars within violins indicate mean ± SEM.

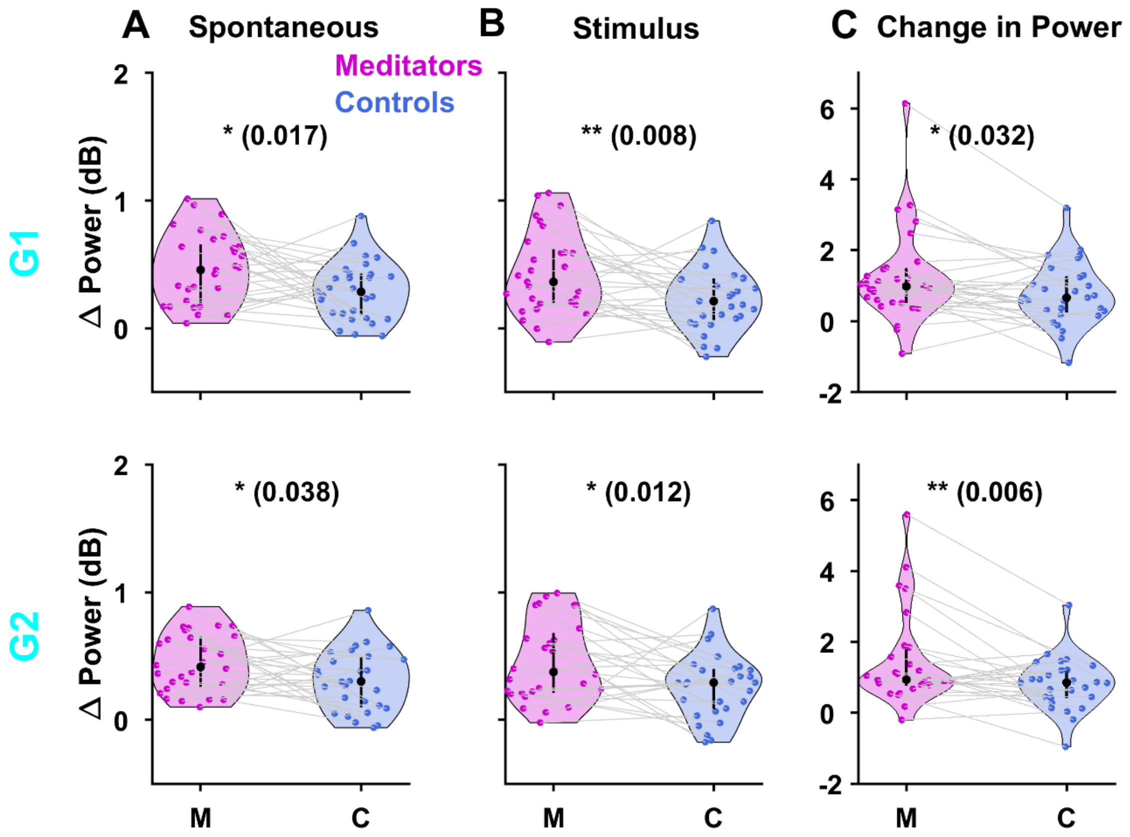

Supplementary Figure 4. Meditators show higher spontaneous, stimulus, and stimulus-induced gamma power before and after meditation. Violin plots showing broadband gamma power (30–80 Hz) in the occipital electrode group for meditators (magenta) and controls (blue) during the pre-meditation (G1; top row) and post-meditation (G2; bottom row) gamma protocols. Each dot represents an individual participant, with grey lines connecting age- and gender-matched pairs. **(A)** Spontaneous (baseline) power, **(B)** stimulus-period power, and **(C)** change in power (stimulus – baseline, in dB). Meditators exhibited significantly higher gamma power than controls across all epochs in both G1 and G2, with stronger effects after meditation. Significance levels correspond to paired t-tests (* $p < 0.05$; ** $p < 0.01$), and exact p-values are shown in parentheses. Error bars denote mean ± SEM within violins.

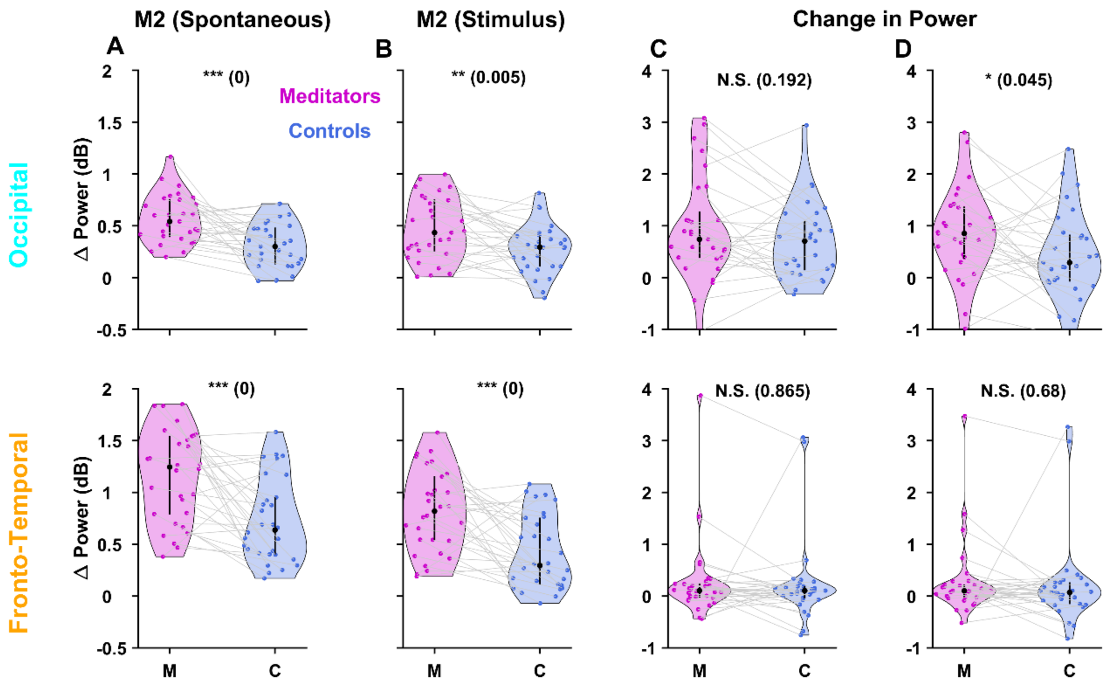

Supplementary Figure 5. Stimulus-induced and meditation-induced gamma co-exist during meditation with visual stimulation (M2). Violin plots showing gamma power in occipital (top row, cyan labels) and fronto-temporal (bottom row, orange labels) electrode groups for meditators (magenta) and controls (blue). (**A**) Power during spontaneous (baseline) periods, (**B**) power during the stimulus period, and (C–D) stimulus-induced change in power (stimulus – baseline, in dB) computed for the (**C**) traditional slow-gamma range (24–34 Hz) and (**D**) slightly lower range (20–30 Hz). Meditators exhibited significantly higher broadband gamma power than controls during both spontaneous and stimulus periods across regions, but the change in stimulus-induced gamma reached significance only when computed in the 20–30 Hz range (D). Each dot represents an individual participant, with grey lines connecting age- and gender-matched pairs. Statistical significance was assessed using paired t-tests (*p < 0.05; **p < 0.01; ***p < 0.005; N.S.: non-significant), with exact p-values shown in parentheses. Error bars within violins indicate mean ± SEM.

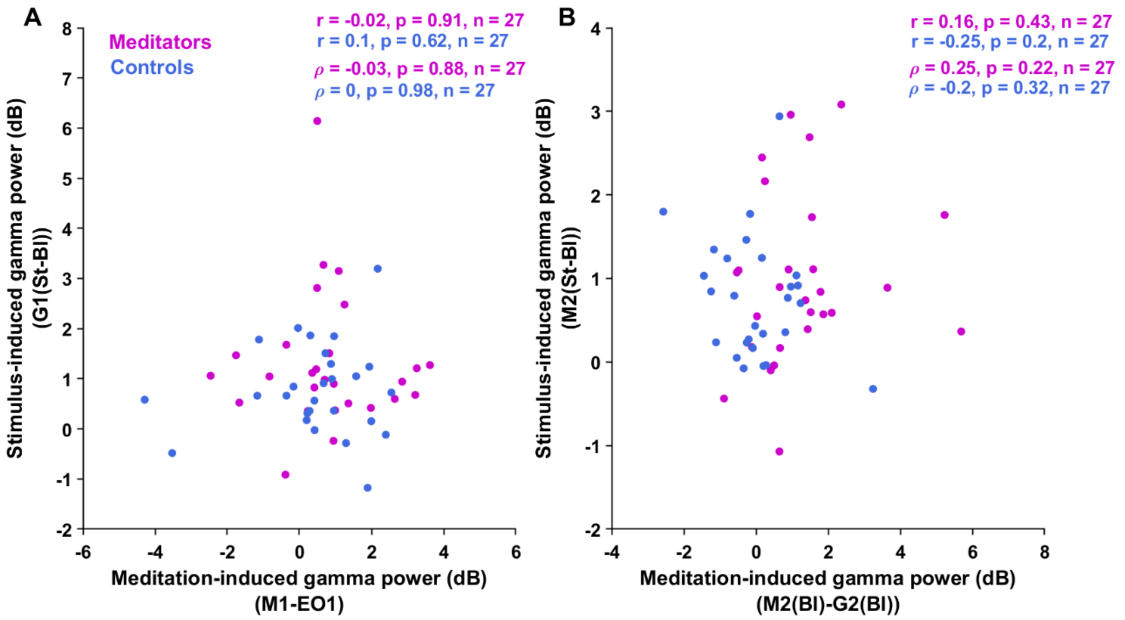

Supplementary Figure 6. Scatter plots showing the comparison of **(A)** meditation-induced gamma power (30-80 Hz) during M1 compared to EO1 versus stimulus-induced gamma power (24-34 Hz) during G1 stimulus compared to G1 spontaneous period **(B)** meditation-induced broadband gamma power (30-80 Hz) during M2 spontaneous compared to G2 spontaneous period vs stimulus-induced gamma power (24-34 Hz) during M2 stimulus compared to M2 spontaneous periods, for meditators (pink dots) and controls (blue dots) for the occipital electrode group. The corresponding parameters for the correlation analysis are shown at the top of each subplot for both the participant groups. Here, r denotes the Pearson correlation coefficient, whereas ρ denotes the Spearman correlation coefficient.

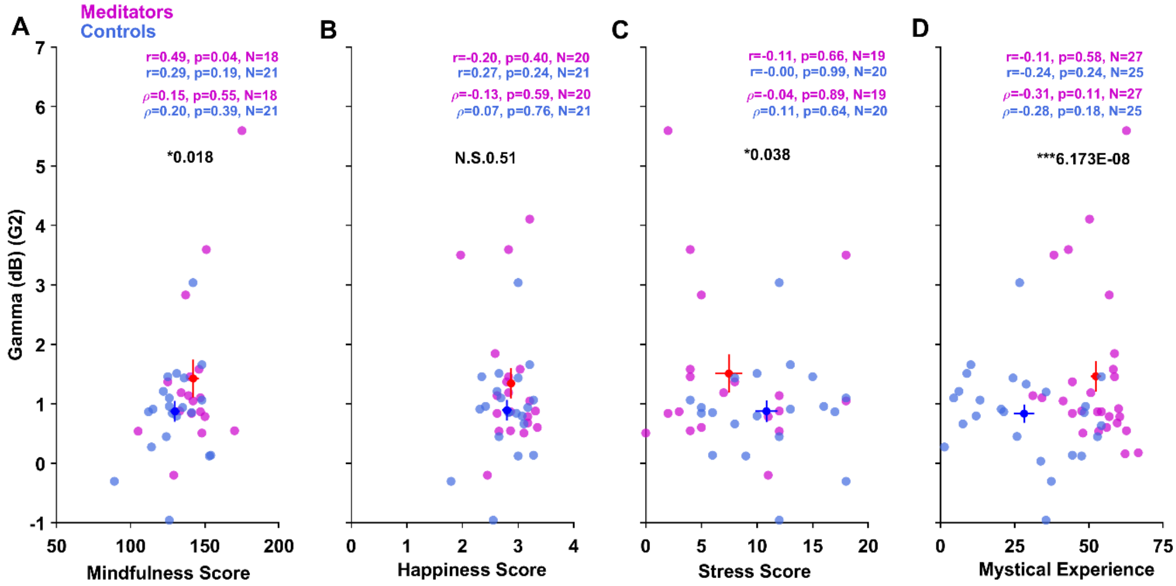

Supplementary Figure 7. Scatter plots show change in gamma power (in the G2 condition) with (**A)** Mindfulness Score **(B)** Happiness Score, (**C)** Stress Score and **(D)** Mystical Experience for meditators (pink dots) and controls (blue dots) for the occipital electrode group. The corresponding parameters for the correlation analysis are shown at the top of each subplot for both the participant groups. In each panel, mean scores for meditators (red dot) and controls (dark blue dot) are also shown. P-values for the t-tests between the mean scores of both the groups are shown at each panel. * Indicate values were less than 0.05 and *** indicate values were less than 0.001. N.S. indicates non-significance. r and ***ρ*** represent Pearson and Spearman correlation coefficients respectively.

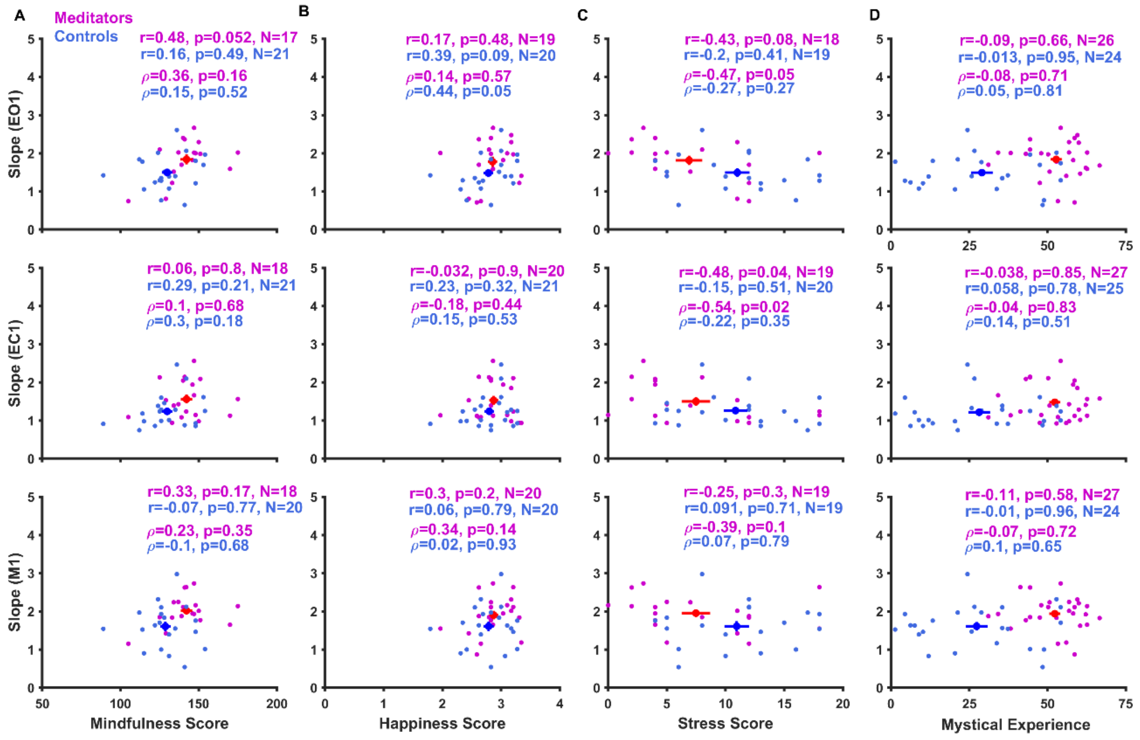

Supplementary Figure 8: Scatter plots show slopes with (**A**) Mindfulness Score **(B)** Happiness Score, (**C)** Stress Score and **(D)** Mystical Experience for EO1 (top), EC1 (middle) and M1 (bottom). Details are same as supplementary figure 7. r and ***ρ*** represent Pearson and Spearman correlation coefficients respectively.
